# Distinct functions of AKT isoforms in breast cancer: a comprehensive review

**DOI:** 10.1186/s12964-019-0450-3

**Published:** 2019-11-21

**Authors:** Nico Hinz, Manfred Jücker

**Affiliations:** Institute of Biochemistry and Signal Transduction, University Medical Center Hamburg-Eppendorf, Martinistraße 52, 20246 Hamburg, Germany

**Keywords:** AKT, Protein kinase B, Isoforms, Breast cancer, PI3K/AKT signaling

## Abstract

**Background:**

AKT, also known as protein kinase B, is a key element of the PI3K/AKT signaling pathway. Moreover, AKT regulates the hallmarks of cancer, e.g. tumor growth, survival and invasiveness of tumor cells. After AKT was discovered in the early 1990s, further studies revealed that there are three different AKT isoforms, namely AKT1, AKT2 and AKT3. Despite their high similarity of 80%, the distinct AKT isoforms exert non-redundant, partly even opposing effects under physiological and pathological conditions. Breast cancer as the most common cancer entity in women, frequently shows alterations of the PI3K/AKT signaling.

**Main content:**

A plethora of studies addressed the impact of AKT isoforms on tumor growth, metastasis and angiogenesis of breast cancer as well as on therapy response and overall survival in patients. Therefore, this review aimed to give a comprehensive overview about the isoform-specific effects of AKT in breast cancer and to summarize known downstream and upstream mechanisms. Taking account of conflicting findings among the studies, the majority of the studies reported a tumor initiating role of AKT1, whereas AKT2 is mainly responsible for tumor progression and metastasis. In detail, AKT1 increases cell proliferation through cell cycle proteins like p21, p27 and cyclin D1 and impairs apoptosis e.g. via p53. On the downside AKT1 decreases migration of breast cancer cells, for instance by regulating TSC2, palladin and EMT-proteins. However, AKT2 promotes migration and invasion most notably through regulation of β-integrins, EMT-proteins and F-actin. Whilst AKT3 is associated with a negative ER-status, findings about the role of AKT3 in regulation of the key properties of breast cancer are sparse. Accordingly, AKT1 is mutated and AKT2 is amplified in some cases of breast cancer and AKT isoforms are associated with overall survival and therapy response in an isoform-specific manner.

**Conclusions:**

Although there are several discussed hypotheses how isoform specificity is achieved, the mechanisms behind the isoform-specific effects remain mostly unrevealed. As a consequence, further effort is necessary to achieve deeper insights into an isoform-specific AKT signaling in breast cancer and the mechanism behind it.

## Background

According to the cancer statistics, breast cancer poses the most common cancer entity in women and causes the second highest number of death by neoplasia after lung cancer [1]. Although the mortality for breast cancer decreased by 40% from 1989 to 2016 [1], formation of metastasis e.g. in the bone impairs prognosis of breast cancer and causes the high mortality rate [2, 3]. Breast cancer preferably metastasizes into lung, pleura, liver, bone and adrenal glands [4].

Hanahan and Weinberg reported their hallmarks of cancer in 2000 and suggest following properties as important milestones of tumor development: persistent cell proliferation e.g. through independence from growth signals, bypassing suppression of growth, resistance against apoptosis, immortalization of the cell, promotion of angiogenesis and induction of invasion and metastasis. In 2011 they added the modification of metabolism in cancer cells as another important part of cancer development [5, 6]. The multistep process of metastasis was further characterized by Gupta and Massagué and is composed of aggressive and invasive phenotype of cancer cells, detachment, intravasation, circulation, homing, adhesion, extravasation and colonization [7].

Since AKT, also known as protein kinase B, is linked to and regulates many of the cancer hallmarks and the metastatic cascade in breast cancer [8–11], much effort was made to develop targeted therapy for AKT signaling in breast cancer [12–15]. Furthermore, AKT seems to be a reasonable target for cancer therapy on the grounds that the PI3K/AKT signaling pathway is frequently dysregulated in up to 70% of human breast cancer [16] and upregulation of AKT in cancer is associated with overall poor prognosis [17]. However, there is growing evidence that the different isoforms AKT1, AKT2 and AKT3 have non-redundant and partly opposing effects in tumorigenesis, making pan-AKT inhibition in breast cancer inappropriate. Long-time lacking awareness for the isoform-specific effects in breast cancer and unavailability of isoform-specific inhibitors and antibodies delayed the investigations of isoform-specific effects in breast cancer and other cancers. In the last years it was possible to close the gap in knowledge more and more by using isoform-specific knockdown or overexpressing vectors in vitro and in mouse models [18]. Hence, we will outline the isoform-specific effects of AKT in breast cancer in vitro and in vivo influencing the hall marks of cancer and the impact of AKT-isoforms on clinical parameters. Afterwards, we will discuss the consensus and differences amongst the studies, possible mechanisms of isoform specificity and the clinical implications of the findings.

## The AKT signaling pathway

The serine/threonine kinase AKT, also known as PKB, was first cloned simultaneously by three laboratories [19–21] after Staal et al. discovered the v-AKT proto-oncogene, a viral homolog in a thymic lymphoma of an AKR mouse [22]. AKT belongs to the ACG family and consists of an N-terminal PH-domain, a linker region, a catalytic domain and a C-terminal regulatory domain [23].

The PI3K-AKT signaling pathway is initiated by binding of growth factors like EGF, IGF-1, heregulin or PDGF to their receptor tyrosine kinases and leads to autophosphorylation of the RTKs [24–27]. PI3K class I, composed of the regulatory subunit p85 and the catalytic subunit p110 [28], is now activated and converts PI(4,5)P_2_ to PI(3,4,5)P_3_ at the plasma membrane of the cell [29, 30]. PI(3,4,5)P_3_ in turn serves as a binding site for the PH-domain of AKT, causing a conformational change [31] and the phosphorylation of AKT at T308 by PDK1 [27, 32]. For fully activation AKT needs to be phosphorylated at S473 in particular by mTORC2 [27], but also by other kinases like DNA-PK [33], PKCγ [34], IKK [35] or by autophosphorylation of AKT [36]. Inactivation of AKT is indirectly done by PTEN due to dephosphorylation of PI(3,4,5)P_3_ to PI(4,5)P_2_ [37] and by SHIP through conversion of PI(3,4,5)P_3_ to PI(3,4)P_2_ [38] or by PHLPP [39, 40] and PP2A [41] which directly dephosphorylates AKT.

After activation of AKT, the kinase dissociates from the plasma membrane and translocates to cytoplasm and nucleus to phosphorylate and activate its substrates [42, 43]. AKT as a basophilically-directed kinase phosphorylates its substrates in a sequence specific context: arginine at residue − 3 relative to the serine/threonine phosphorylation site, in most cases also an arginine at residue − 5, a hydrophobic motif at residue + 1 and a proline at residue + 2 [44]. The catalytic activity of AKT spreads over more than 100 substrates that are involved in metabolism, proliferation, apoptosis, protein expression, migration and much more [9, 45]. For instance, AKT causes the translocation of GLUT4 via phosphorylation of AS160 as well as PIKfyve and therefore increases glucose uptake [46–48]. Cell proliferation is positively regulated through prevention of cell cycle stimulating cyclin D1 degradation via inhibitory phosphorylation of GSK3 by AKT [49, 50]. Furthermore, AKT directly inhibits the cell cycle inhibitors p21^waf1^ and p27^kip1^ [51, 52] and induces Mdm2-mediated inhibition of the tumor suppressor p53 [53]. Inhibitory phosphorylation of BAD [54], pro-caspase 9 [55] and pro-apoptotic transcription factors of the FOXO-family by AKT [56] prevents the cell from apoptosis. AKT-mediated induction of NF_K_B-dependent transcription of anti-apoptotic proteins like Bcl-XL [57] is also involved in regulating cell survival. In addition, cell migration and invasion is increased e.g. due to phosphorylation of the pro-migratory actin-binding protein Girdin [58] and induction of MMP2 and MMP9 by AKT [59, 60]. Moreover, AKT regulates protein expression through activation of the mTORC1/p70S6K/S6 axis and induction of eIF4E via inhibition of the negative regulator TSC2 and PRAS40 [61–63]. As a result, AKT in general is involved in regulating proliferation, survival, migration and metastasis of breast cancer [11, 64–68].

Thus, it is not surprising that the complete PI3K/AKT signaling pathway is frequently dysregulated in human cancer, especially in breast cancer. The most common dysregulation represents mutations of the PIK3CA itself [69–71]. Further frequently occurring alterations of the PI3K/AKT signaling are inactivation of PTEN [71, 72], amplification of HER2 [73] and more rarely mutations or amplifications of AKT itself [74, 75]. The HER2 status is involved in the subtyping of breast cancer, in detail composed of luminal A (HER-, ER+, low proliferation), luminal B (HER+ or -, ER+, high proliferation), HER2-type (HER2+, ER+ or -) and basal like (triple negative breast cancer) [76]. Several studies reported an association of AKT with breast cancer initiation [77], prognosis [16, 78], metastasis [79] and resistance to chemotherapy [80] as well as improved hormonal therapy [17, 81, 82]. Surprisingly, the studies disclose partly opposing results of AKT [83, 84]. On that basis, the challenge began to develop therapeutics that target the PI3K/AKT signaling like the PI3K inhibitor LY294002 [85], inhibitors for mTOR [86] and pan-AKT inhibitors like MK2206 [87–90] or perifosine [91]. There is further rationale to target AKT because of induction of apoptosis by kinase-dead AKT in tumor cells or by inhibition of AKT activity in tumor cells with a high AKT activity [92].

## The three AKT isoforms

During investigations of the AKT signaling more and more evidence raised that the three isoforms AKT1 (PKBα), AKT2 (PKBβ) and AKT3 (PKBγ) exert distinct and partly even opposing effects in cancer and physiologically. After the detection of AKT1 and AKT2 by Jones et al. [21, 93], AKT3 was discovered a few years later [94, 95]. AKT3 is constituted of the two distinct splicing variants AKT3 + S472 and AKT3-S472, the latter one lacking the phosphorylation site on position S472 [96]. Different genes are encoding for the three isoforms: AKT1 is located at 14q32 [97], AKT2 at 19q13.1–13.2 [98] and AKT3 at 1q44 [99]. The AKT isoforms have about 80% similarity in the amino acid structure [11, 100], in detail AKT1 and AKT2 share 82% similarity at the amino acid level, AKT2 and AKT3 77% and AKT1 and AKT3 83% [101]. The highest similarity is located in the catalytic domain with 87 to 90% and the greatest diversity is located in the linker region with 17 to 46% similarity [92]. Although several differences exist in the structure of the isoforms, they all possess similar phosphorylation sites: T308 and S473, T309 and S474 as well as T305 and S472 for AKT1, AKT2 and AKT3, respectively [95]. AKT1 and AKT2 are expressed ubiquitously with a predominant expression of AKT2 in insulin-responsive cells, whereas expression of AKT3 is limited most notably to neurons and the testes of mice [94].

First findings about non-redundant isoform-specific effects in cells originated from mouse knockout models. AKT1 knockout mice show a decreased body weight, increased apoptosis in thymocytes and testes and enhanced neonatal mortality. This suggests that AKT1 is important for physiological placental development as well as cell proliferation and growth [102]. Knockout of AKT2 in a mouse model leads to a diabetes-like phenotype through peripheral insulin resistance and impaired glucose uptake into the cell, confirming a pivotal role of AKT2 in glucose homeostasis [103, 104]. Mice lacking AKT3 display reduced brain size, indicating AKT3 as the important isoform for physiological brain development [105, 106].

In addition to these data, double knockout of AKT isoforms in mice shows some evidence for overlapping functions of the distinct isoforms. Lack of AKT1 and AKT2 results in neonatal death [107], furthermore knockout of AKT1 and AKT3 leads to perinatal mortality [108]. However, simultaneously knockout of AKT2 and AKT3 causes reduction of body weight and insulin resistance but is not accompanied by lethality [109]. Moreover, mosaic activating mutations of isoforms generate distinct phenotypes in human, e.g. hypoglycemia in mosaic activating mutations of AKT2 or an enlarged cerebral hemisphere due to activating mutations of AKT3 [110].

Together with the fact that activation of the PI3K/AKT signaling can be associated with a good outcome in breast cancer [111] and a decrease in cell migration in some studies [112, 113], inhibition of all AKT isoforms might cause unwanted effects or higher toxicity like perturbations in the glucose homeostasis. As a result, investigations of isoform specificity in breast cancer and other cancers began.

## AKT isoform specificity in physiological mammary development

Mammary gland involution is an important step to remove mammary epithelium, if its lactating function is no longer required after lactation. Apoptosis plays a crucial role during this process and enables the structural remodeling of the mammary gland [114, 115]. AKT in general delays involution in mammary glands of MMTV-AKT transgenic mice due to suppressed apoptosis and prolonged expression of TIMP-1, an inhibitor of MMPs. These findings indicate that AKT has an important role in physiological involution of mammary glands [116, 117].

Subsequently, Ackler et al. investigated whether the involution of the mammary gland is altered in MMTV-AKT1 transgenic mice. AKT1 expression in the mammary gland delays involution via elevated levels of cyclin D1. This effect is suggested to originate from phosphorylation of GSK3 and increased phosphorylation of BAD. AKT1-MMTV mice do not show sustained dysplasia or neoplasia in the mammary gland, proposing AKT1 has no transforming ability [118, 119]. AKT1 is further important in the formation of physiological ductal structures, the initiation of lactation and a change in the lipid and glucose metabolisms during lactation [120, 121]. Accordingly, lack of AKT1 in mammary epithelial cells impairs lactation and accelerates involution, whereas knockdown of AKT2 induces lactation, but decreases involution in the mammary gland [122]. Similar to these findings, AKT2 impairs involution of the mammary gland in MMTV mice through prevention of apoptosis in the mammary cells [123].

In contrast, some studies reported no effect of either AKT1, AKT2 or AKT3 in development and outgrowth of the ductal mammary architecture [124].

## AKT isoform specificity in breast cancer: proliferation and apoptosis in vitro and primary tumor growth in vivo

As mentioned above, AKT in general increases proliferation due to regulation of the cell cycle and prevents the cell from apoptosis by inhibiting pro-apoptotic proteins and promoting anti-apoptotic signaling. For instance, AKT activation in a breast cancer mouse model promotes tumor initiation and progression by decreasing apoptosis through phosphorylation of FOXO and increased levels of Cyclin D1 [117]. Investigating the AKT isoform-specific effects on proliferation, Hutchinson et al. continued their work of 2001. Similar to the group of Dillon et al., they revealed AKT1 as the main isoform mediating the positive effect on tumor initiation and progression in ErbB-2- and PyMT-mediated breast cancer mice, whereas AKT2 has no effect on tumor initiation. The accelerated proliferation is caused by phosphorylation of Rb and elevated levels of cyclin D1 at the post-transcriptional level, possibly through AKT1-mediated inhibition of GSK3. However, neither activation of AKT1 nor AKT2 is able to generate tumors in mice without the transforming ErbB2-mediated mammary tumorigenesis [118, 123, 125]. An increase in Cyclin D1 levels upon AKT1 activation was also reported in some other studies [126–128]. Maroulakou et al. confirmed these findings by showing an inhibiting effect of AKT1 ablation on mammary tumor induction and growth in the MMTV-PyMT or -neu mice. This effect is mediated by an impaired proliferation accompanied by lower cyclin D1 levels and increased apoptosis in the AKT1 knockdown. Furthermore, knockdown of AKT2 in the mice results in enhanced proliferative capacity and tumor growth, whereas ablation of AKT3 has a slight non-significant effect by attenuating tumor growth and induction. The different isoforms are associated with distinct histopathological subtypes of the breast tumor [124]. Riggio et al. reproduced the tumor growth-mediating function of AKT1 in vivo by using AKT1 overexpression [65] and Liu et al. reproduced the pro-proliferative and anti-apoptotic ability of AKT1 in vitro [129].

Besides the oncogenic character of AKT1, AKT3 was also identified as an oncogene, as knockdown of AKT1 and AKT3 impairs proliferation of HER2-positive cells [130]. These findings were mainly reproduced in TNBC in which ablation of AKT1 and AKT3 decreases proliferation in vitro and tumor growth in vivo through a lack of activating interaction with DNA-PKcs. Interestingly, knockdown of AKT2 had no effect on proliferation in vitro in this study, whereas tumor growth in vivo was higher [131]. Moreover, AKT1 promotes spheroid growth [132]. Whereas all three isoforms were shown to be important for formation of spheroids, AKT2 is the specific isoform maintaining the architecture of the spheroids. Withal, this study observed that PTEN deficient cells obtain tumorigenicity through specific signaling via AKT2 [133].

In addition to the Rb-, cyclin D1- and DNA-PK-mediated increase of proliferation by AKT1, the study of Héron-Milhavet et al. provides solid understanding of the interaction between AKT and p21 by using human fibroblasts. AKT1 increases cell proliferation by causing delocalization of p21 out of the nucleus via T154 phosphorylation and therefore disinhibition of G1-S-transition. AKT2, by contrast, induces cell differentiation and cell cycle exit through localization of p21 to the nucleus by directly binding to it and therefore preventing AKT1-mediated phosphorylation. Moreover, phosphorylation of the cyclin-dependent kinase inhibitor p21 by AKT1 leads to release of CDK2 and therefore higher levels of cyclin A which contributes to cell cycle progression [134]. In addition, AKT1 phosphorylates Skp2 leading to its stabilization and cytoplasmic translocation and as a consequence Skp2 causes a destruction of the cyclin-dependent kinase inhibitor p27 [135]. Similar to the effect of AKT2 on nuclear translocation of p21, overexpression of AKT2, but not AKT1, accelerates p27 stabilization and translocation to the nucleus. High levels of AKT2 inhibit CDK2 as an inductor of cell cycle and inhibit cell proliferation [126]. Ju et al. showed an abundance of p21 and p27 that is induced by AKT1 and promotes ErbB2-dependent tumor growth in vivo. These findings contravene the statements of all other studies about the function of p21 and p27 as inhibitors of the cell cycle [136].

Knockdown of AKT2 in the study of Santi et al. caused a decreased proliferation in the same TNBC that Yang et al. have used. Cells lacking AKT2 exhibit downregulation of CDK2 and cyclin D as well as upregulation of p27, resulting in a cell cycle arrest. In addition, AKT2 ablation leads to mitochondrial autophagy through the discrepancy of increased mitochondrial biogenesis via enhanced PGC-1α activity and decreased protein expression via downregulated p70S6K [128]. This suppression of proliferation by AKT2 knockdown was confirmed by Wang et al. [137]. Downregulation of AKT2 by Metformin-stimulated upregulation of miR-200c was discovered as another possible mechanism [138].

Notably, AKT2 is supposed to regulate the other AKT isoforms under hypoxic conditions and therefore serves as a master regulator of AKT activity. Hypoxia induces expression of AKT2, but not of AKT1 or AKT3, and AKT2 in turn upregulates miR-21 via activation of NF_K_B and CREB. The transcription factors bind to the miR-21 promotor and lead to acetylation of histone structures H3K9. MiR-21 in turn suppresses the protein levels of PTEN, pro-apoptotic PDCD4 and Sprouty1. Through the inhibition of PTEN, AKT2 is able to activate all three AKT isoforms in a hypoxic environment. As a consequence of the AKT activation, the cells acquire a higher survival under hypoxic environment and thus AKT2 promotes tumorigenic properties of breast cancer cells [139]. WDR26 is a scaffolding protein that fosters the formation of a complex, containing PI3Kβ, Gβγ and AKT2. Formation of this complex leads to specific activation of AKT2 by GPCRs via the PI3Kβ isoform, a mechanism that is also involved in PTEN deficiency. AKT2 promoted breast cancer cell growth after stimulation of GPCR e.g. by SDF1α in this study [140].

S6 was identified as another potential mediator for AKT1-specific induction of proliferation and in vivo tumor growth. AKT2 has no effect on S6 and therefore only slightly decreases proliferation in vitro, without affecting tumor growth in vivo in this study [127]. The amount of the tumor suppressor p53 is higher in low-proliferative cells lacking AKT1 [141]. Overexpression of AKT1, but not of AKT3, results in enhanced phosphorylation of the tumor suppressor SIRT6 at S338. As a consequence, MDM-2 mediated proteasomal degradation of SIRT6 is accelerated and therefore proliferation and tumor growth are elevated [142]. AKT1 overexpression retains BRCA1 and RAD51 in the cytoplasm, resulting in a heightened genomic instability through an impaired DAN-repair by homologous recombination which maintains at a level sufficient for cell proliferation. This generates a BRCA1 deficient-like phenotype in breast cancer, whereupon AKT1 is necessary for the BRCA1-associated breast cancer cell proliferation [121, 143]. PIPP is a possible suppressor of AKT1-mediated enhancement in proliferation, survival and tumor growth by dephosphorylation of PI(3,4,5)P_3_ to PI(3,4)P_2_ and therefore impaired AKT1 activation [144]. Another protein that can act upstream and suppresses the tumor growth-stimulating AKT1 is miR-409-3p [145]. Par1 was identified as an upstream activator of AKT1 that is activated in turn by MMP1 [146].

In opposition to the studies confirming distinct roles of AKT1 and AKT2 in cell proliferation and tumor growth, Watson and Moorehead attribute AKT1 knockdown as well as AKT2 knockdown to a suppressing effect on tumor initiation and growth of an IGF1R-positive breast cancer mouse model. Proliferation of breast cancer cells is diminished in both knockdowns [147]. Irie et al. confirmed these findings by showing a decrease in proliferation in vitro in IGF-1R-positive mammary epithelial cells at basal levels as well as upon IGF-1 stimulation for both knockdowns [148]. Ablation of AKT1, AKT2 or AKT3 reduced proliferation in the study of Chin et al. [133]. Gargini et al. reported a suppression of proliferation in breast cancer cells with either AKT1 or AKT2 knockdown through cell cycle arrest. Additionally, knockdown of AKT1 increases apoptosis by enhancing the protein level of pro-apoptotic Bim via regulation of FoxO3 [149].

In total contrast Yang et al. uncovered that overexpression of AKT1, but not of AKT2, can inhibit proliferation by phosphorylating Raf at S259 and therefore inhibiting the pro-proliferative Raf/MEK/ERK signaling in TNBC [126]. Further conflicting results were published e.g. missing effects on proliferation of TNBC in knockdown of any AKT isoform [150] and also unaltered tumor growth in vivo in AKT1 or AKT2 knockdown [151]. Choi et al. were not able to detect an effect of AKT1 on proliferation either [152].

The role of AKT3 in proliferation, apoptosis and tumor growth was examined next. Overexpression of AKT3 in ER-positive breast cancer cells induces estrogen-independent growth in vitro and in vivo that can be inhibited by estrogen supplementation. The reason for this is a decrease in ER levels after AKT3 overexpression, but however the phosphorylation of ER at S167 rises without inducing activity [153]. Ablation of AKT3 in TNBC decreases proliferation [128], whereas another study denied any effect on proliferation of AKT3 [154]. Spheroid growth in vitro and tumor growth in vivo are suppressed in TNBC cells lacking AKT3 via upregulation of p27 [132, 151]. The tumor-suppressor miR-433 directly targets AKT3 and hence attenuates proliferation, cell viability and survival; the latter probably through downregulation of Bcl-2 and upregulation of BAX [155]. Additionally, another micro RNA, miR-29b, targets and inhibits AKT3 and therefore causes reduction in proliferation and survival. Overexpression of AKT3 in comparison leads to downregulation of p53, p21 and p27 and upregulation of Cyclin D1, Bcl2 and XIAP [156]. Suyama et al. investigated the role of AKT3 in a more differentiated manner, considering the two different splice variants AKT3 + S472 and AKT3-S472; the latter without the S472 phosphorylation site. Knockdown of AKT3-S472 in TNBC displays an enhanced tumor growth in vivo by downregulating Bim via activation of the MAPK/ERK pathway and therefore inhibition of BAX [96].

Astonishingly, in inflammatory breast cancer AKT3, but not AKT1 or AKT2, increases proliferation and decreases apoptosis [157].

## AKT isoform specificity in breast cancer: migration and invasion in vitro and metastasis in vivo

Migration and invasion are striking steps in the metastatic process of breast cancer. Thus, also migration, invasion and metastasis are regulated by AKT in an isoform-specific manner. Hutchinson et al. first reported a decrease in lung metastasis in the AKT1 activated breast cancer mouse model through an accelerated differentiation of the mammary tumor cells and therefore loss of their metastatic potential. The effect of AKT1 is hypothesized to be a consequence of regulation of the basal membrane components, e.g. laminins or collagens that counteracts a metastatic-typical degradation of the extracellular matrix [125]. In another study AKT1 only impairs metastasis formation in MMTV-ErbB2 mice but has no effect in the MMTV-PyMT mice. An enhanced abundance of the anti-metastatic ERα in the AKT1 activated cells, especially in the nucleus, serves as a possible explanation for the anti-metastatic abilities of AKT1 in this study [123]. Likewise, wound healing assays in vitro also identified AKT1 as an anti-migratory isoform [126]. A couple of studies confirmed the anti-metastatic and anti-migratory ability of AKT1 independent of the breast cancer subtype [113, 119, 151, 154].

Several studies address the mechanisms of the AKT1-mediated reduction in migration and metastasis. The isoform-specific substrate palladin was detected by Chin and Toker in 2010 as an actin-binding protein that is specifically phosphorylated at S507 and therefore activated by AKT1. The isoform specificity of palladin is mediated exclusively by the linker region of AKT1. Phosphorylation of palladin was observed in a PI3K-dependent manner after stimulation with EGF or IGF-1 as well as through the PIK3CA mutations H1047R and E545K. The active state of palladin reduces migration and invasion due to an augmented actin bundling and a decreased formation of invadopodia. Although AKT 2 does not have the ability to phosphorylate palladin directly, AKT 2, but not AKT1, increases expression of palladin by regulating protein stability and protein transcription. Remarkably, increased levels of palladin are associated with invasive breast cancer [158–160].

AKT1 blocks migration and invasion of breast cancer cells through an inactivating phosphorylation of GSK3 and therefore a HDM2-mediated proteasomal degradation of the pro-migratory transcription factor NFAT1 [112, 161]. Liu et al. evolved a model in which AKT1 induces the 14–3-3-mediated proteolytic degradation of TSC2. Lower levels of activated TSC2 diminish the activation of the pro-migratory Rho GTPase and therefore cause dysregulated focal adhesions and actin-cytoskeleton e.g. lower stress fiber formation [129]. Riggio et al. detected an increase in invasion and migration by AKT1 knockdown through a lack of inhibition of β1-integrin expression and FAK phosphorylation. Furthermore, AKT1 mediates invasiveness by regulating MMP9 and E-cadherin. Hence, the number of lung metastasis in mice is elevated by an AKT1 knockdown. Moreover, the mouse mammary tumors of AKT1 knockdown cells exhibit a more spindle-shaped morphology, indicating a higher invasiveness [127].

TIS21 upregulates the activation of AKT1 which in turn downregulates expression of NOX4 via downregulation of its transcription factor Sp1. The lack of NOX4 results in attenuated levels of ROS, consequently a decreased expression of mDia1, 2 and 3 and therefore inhibition of invasion and migration through impaired F-actin-polymerization and decreased formation of stress fiber and invadopodia [152]. PIPP dephosphorylates PI(3,4,5)P_3_ to PI(3,4)P_2_ and consequently suppresses AKT1-mediated impairment of migration, invasion, chemotaxis and metastasis. This can provide a mechanism behind the AKT1-dependent regulation of NFAT1, TSC2 and Mmp2 [144]. In addition, IGF-1 and EGF stimulation as well as basal conditions in the knockdown of AKT1, but not AKT2, in mammary epithelial cells MCF10A lead to an enhanced EMT. Downregulation of E-cadherin, upregulation of N-cadherin and emergence of a spindle-shaped cell morphology indicate the EMT and occur through an enhanced ERK activation. AKT1 inhibits the ERK-signaling in an isoform-specific manner [148, 162]. Upregulation of the pro-migratory transcription factor β-catenin and its nuclear translocation through activation of EGFR and ERK signaling are other consequences of AKT1 knockdown [113]. Ablation of AKT1, but not of AKT2, also promotes EMT of breast cancer cells especially after TGF-β stimulation by decreasing the amounts of the miR-200 family. Consequently, reduced levels of miR-200 increase the E-cadherin suppressors Zeb1 and Zeb2 in an AKT2-dependent manner [163]. MiR-409-3p is an additional AKT1-specific upstream regulator that suppresses the total AKT1 protein amount in breast cancer cells [145].

In contrast to the anti-metastatic function of AKT1, Irie et al. noted a decrease in migration of EGF-stimulated AKT2 knockdown mammary epithelial cells due to a reduced vimentin expression. Supplementary, knockdown of AKT2 reduces the higher migration in AKT1 knockdown mammary epithelial cells, suggesting AKT2 mediates migration as the predominant isoform [148]. To further determine the role of AKT2 in enhancing migration and metastasis Dillon et al. investigated MMTV-PyMT and MMTV-ErbB2 mice with ectopically expressed AKT2. They observed an AKT2-mediated formation of metastases in both mouse models. In addition, AKT2, but not AKT1, overexpressing clones exhibit an accelerated invasion in vitro. In line, highly invasive clones of a breast cancer cell line show elevated levels of AKT2 expression and pAKT2 [123].

A couple of studies confirmed the pro-migratory role of AKT2 [151, 157, 164–166] and dealt with possible mechanisms behind the crucial role of AKT2 in breast cancer migration, invasion and metastasis. AKT2, but not AKT1 or AKT3, enhances an integrin β1-mediated attachment to and an invasion through collagen IV and to a minor degree through laminin in vitro and in vivo. Hence, AKT2 is predominantly localized at the basal part of the cell that exhibits cell-matrix-interaction. This higher extent of invasion in AKT2 overexpressing cells is still dependent on PI3K activity. Furthermore, the post-invasion survival of AKT2 overexpressing breast cancer cells is augmented and contributes to the increased metastatic potency in vivo. Interestingly, non-transformed mammary epithelial cells do not display an invasive phenotype, not even when AKT2 is overexpressed [167]. AKT2 directly interacts with PKCζ after EGF-stimulation and therefore activates actin-polymerizing LIMK/Cofilin axis and adhesion associated β1-integrin. This explains the enhanced chemotaxis to EGF that is mediated by AKT2 [137]. Knockdown of AKT2 suppresses invasion and migration due to lower levels of F-actin and vimentin. Consequently, lung metastasis is also elevated in breast cancer cells overexpressing AKT2 [127].

The transcription factor Twist specifically upregulates AKT2 expression by transactivation of its promotor and therefore causes EMT-mediated migration, invasion and metastasis as an early process in breast cancer progression [164, 168]. Moreover, the PIK3CA mutation H1047R, but not E545K, specifically activates AKT2 and consequently promotes invasion and migration in mammary epithelial cells [169]. Stimulation of GPCRs e.g. by LPA or SDF1α leads to specific activation of AKT2 via complex formation with PI3Kβ which is promoted by WDR26. Thereby, activated AKT2 is also inducing chemotaxis towards LPA or SDF1α [140]. Metformin can raise expression of miR-200c which in turn decreases AKT2-dependent migration and invasion [138].

A possible link between the effects of AKT1 and AKT2 was revealed by Li et al. AKT1 suppresses invasion, migration and metastasis by phosphorylating Twist1 at S42, T121 and S123 and therefore increases degradation of Twist1. Twist1 typically increases pro-migratory EMT and mediates its effect at least in part through upregulation of AKT2 as mentioned above. Surprisingly, AKT2 itself phosphorylates Twist1 at S42 without affecting its degradation [170].

In total contrast, AKT2 overexpressing cells exhibited an anti-migratory phenotype in the study of Yang et al., although most studies point to an enhancing effect of AKT2 on migration, invasion and metastasis [126].

There are some studies astonishingly reporting a pro-migratory and pro-metastatic effect of AKT1. Ju et al. revealed an induced phosphorylation of TSC2, induced cortical F-actin as well as alteration of cytoskeleton components like paxillin and ezrin-radixin-moesin by AKT1 and therefore observed an impaired migration in AKT1 knockdown breast cancer cells. These in vitro findings were supplemented by a reduced metastasis in vivo in AKT1 knockdown cells. Furthermore, AKT1 shows an induced expression of pro-migratory secreted factors like MIPγ, SDF-1 and CXCL-16 and partly even the corresponding receptors [136]. The findings of Ju et al. were supported by results, showing augmented invasion in vitro and metastasis of mammary epithelial cells in vivo by AKT1. These findings are based on an induced ECM degradation by an AKT1-mediated enhancement of MMP2 levels via posttranscriptional stabilization, perhaps through inhibition of GSK3-dependent degradation [59]. An upstream regulator of AKT1 is the protein PAR1 that can be activated by MMP1 and in turn activates AKT1-mediated migration, invasion and metastasis [146]. In concert, AKT1 knockdown in the MMTV-PyMT, but not in the MMTV-neu, mouse model confirmed a pro-metastatic effect of AKT1. Surprisingly, the AKT1 knockdown breast cancer cells in this study showed a higher invasiveness despite lower metastasis [124].

Hohensee et al. linked the characteristic loss of PTEN in brain-seeking breast cancer cells to an activation of AKT1, whereas the activity of AKT2 and AKT3 remains unchanged. PTEN-mediated suppressed levels of AKT1 activity cause a reduction of migration in general and in co-culture with astrocytes as well as invasion in ex vivo brain slice co-culture. The promoting effect on migration and invasion in cells with higher AKT1 activation depends on an activating crosstalk between astrocytes and the tumor cells via cytokines with autocrine and paracrine effects like BDNF and GM-CSF [171].

There are some studies reporting neither an effect of AKT1 nor AKT2 on migration, metastasis and pro-invasiveness EMT which disagrees with the studies discussed above [65, 147, 150]. Meanwhile, there was no effect of AKT2 on migration or invasion in the study of Choi et al. [152].

Only a few studies paid attention to the role of AKT3 in breast cancer. Grottke et al. investigated the influence of AKT3 on metastasis of TNBC cancer cells in vitro and based on a mouse model. Single knockdown of AKT3 or double knockdown of AKT3 and AKT1 or AKT3 and AKT2 are associated with an increased migration and augmented chemotaxis which seems to be less coordinated. Hence, AKT3 knockdown cells form more metastases in the lung in vivo. The increased migration by a lack of AKT3 is mediated by an upregulation of the pro-migratory protein S100A4 probably via NFAT5. S100A4 is further suggested to mediate EMT and increased activity of MMPs. There is no effect on integrin β1 or Rictor in the AKT3 knockdown tumor cells [150, 151]. In particular, AKT3-S472 suppresses metastasis of TNBCs [96]. Expressions of N-cadherin suppresses expression of AKT3, but not AKT1 or AKT2, and consequently elevates migration [154].

In contrast, Stottrup et al. observed an increase in N-cadherin expression during AKT3 overexpression in AKT inhibitor-resistant cells and therefore increased invasion in the AKT3 overexpressing cells [132]. MiR-29b downregulates AKT3 and therefore caused a suppression of migration and invasion in this study [156].

Investigations of the highly invasive inflammatory breast cancer subtype by Lehman et al. yielded an enhanced migration and invasion through AKT1-mediated activation of RhoC GTPase, whereas AKT2 had no effect. As a result AKT1 levels are increased and AKT2 levels are decreased in inflammatory breast cancer compared to normal breast tissue [157]. Caveolin-1 was identified as a potential specific activator of AKT1 in inflammatory breast cancer [172].

## AKT isoform specificity in breast cancer: angiogenesis and tumor surrounding stroma

Angiogenesis is also influenced by AKT in an isoform-specific manner. AKT1 knockdown in mice causes a decreased VEGF-mediated angiogenesis by affecting the migration of endothelial progenitor cells and the release of NO. Whereas AKT2 does not display such a phenotype [173], AKT3 promotes angiogenesis via VEGF and c-Myc in breast cancer [156]. Impressively, knock down of AKT1 in another mouse model causes an increased vascular density of the mammary tumor [136].

There is some sparse evidence for the importance of the tumor surrounding stroma in breast cancer initiation and progression. Cancer-associated fibroblasts have the ability to increase AKT1 activation in mammary epithelial cells through direct cell-cell-contact and therefore silencing of the tumor suppressor Cystatin M via promotor hypermethylation [174].

## AKT isoform specificity in breast cancer: stem cell phenotype

The AKT isoforms have distinct functions in maintaining the stemness character of breast cancer cells. AKT1 knockdown cells show high levels of vimentin and low levels of E-cadherin, indicating stemness characteristics, whereas AKT2 knockdown cells exhibit the opposite phenotype without stemness characteristics, indicating AKT1 is the critical isoform for promoting the stemness character in vivo [127, 175]. Likewise, the stem cell-like phenotype in AKT1 knockdown breast cancer cells is linked to the EMT caused by low levels of miR-200. As mentioned above small amounts of miR-200 mediate abundance of the E-cadherin suppressors Zeb1 and Zeb2 [163]. On the other hand, AKT1 expression is linked to survival, proliferation and formation of mammospheres formed out of cancer stem cells as well as maintaining the EMT-phenotype with high vimentin and low E-cadherin and FAK expression [65, 176]. Gargini et al. reported that ablation of AKT1 and to a minor degree of AKT2 resulted in a loss of the stem cell phenotype of breast cancer stem cells through the induction of a mesenchymal-epithelial transition by increasing Bim levels via regulation of FoxO3. As a result, mammosphere growth and survival were impaired due to AKT1 or AKT2 knockdown. This was accompanied by an increase in E-cadherin expression and a decrease in expression of vimentin, β-catenin and integrin β1 in AKT1 knockdown cells. Interestingly, AKT2 knockdown leads to an increase in β-catenin expression, a reduction of integrin β1 and has a negligible effect on expression of E-cadherin and vimentin [149].

## AKT isoform specificity in breast cancer: hormone dependency

The AKT isoforms are involved in the regulation of hormone receptors and the hormone-dependency of breast cancer cells. Overexpression of AKT1 in hormone-dependent breast tumors shifts them to a hormone-independent phenotype, including hormone-independent growth, ductal-like differentiated morphology and the corresponding luminal makers like CK8, E-cadherin, laminin-1 and collagen-IV. Estrogen-independent activation of progesterone receptor and ERα through enhanced receptor expression underlies the hormone-independency in AKT1 overexpressing cells [65]. Overexpression of AKT1 increases ERα levels through phosphorylation of the ER at S167 and therefore decreased proteasomal degradation [119, 177]. .Withal, the effect of AKT1 overexpression on transcriptional activity of ER is dose-dependent. Low doses of AKT1 overexpression elevate ERα transcriptional activity, whereas high doses suppress the transcriptional activity of ERα probably through impaired degradation which is necessary for transcriptional activity [177]. In addition, AKT1 mediates the pro-proliferative and pro-survival signals of estrogen and IGF-1 on breast cancer cells [178]. Also, AKT2 promotes the transcriptional activity of ERα in an estrogen-independent manner through phosphorylation of ERα at S167. Moreover, AKT2 mediates the EGF- and IGF1-induced ERα-mediated transcription and AKT2 in turn gets activated by higher ERα levels, suggesting AKT2 can stimulate its own activation [179]. Knockdown of AKT2, but not of AKT1, decreases expression of the ER at the genomic level and diminishes its transcriptional activity, at least in part through an attenuated translocation of FOXO3a out of the nucleus by the AKT2 knockdown. Thus, AKT2 can also cause hormone-independency in breast cancer [180].

## AKT isoform specificity in breast cancer: isoform-specific inhibition in treatment

Since the role of AKT in breast cancer is known, the effect of several pan-AKT inhibitors on tumor growth was examined. For instance, the allosteric pan-AKT inhibitor MK2206 was extensively studied in vitro, in vivo and in first clinical trials either as a monotherapy or in combination with established drugs like the HER2 inhibitor lapatinib [87, 88, 90]. Further pan-AKT inhibitors like perifosine, AZD5363 and Ipatasertib are under clinical investigations for the usage in breast cancer [91, 181]. Because of the growing evidence about the distinct effects of AKT isoforms and notable side effects of pan-AKT inhibitors like hyperglycemia or diarrhea [88, 90], the development of isoform-specific inhibitors seems like a promising approach. In spite of the high homology among the AKT isoforms and therefore the difficulty to develop isoform-specific drugs, Barnett et al. developed isoform specific inhibitors for AKT1 or combinatorial AKT1/2-inhibition. These inhibitors are confirmed to specifically block the phosphorylation and activity of the fitting AKT isoform. The effect of the isoform-specific inhibitors depends on the PH-domain [12]. Treatment with an allosteric AKT1/2-inhibitor that does not affect AKT3 suppresses growth of breast cancer cells that show a dysregulated AKT signaling through PIK3CA mutations or HER2 amplification. Accordingly, breast cancer cells with wildtype PI3K or PTEN expression are resistant to AKT1/2-inhibition. This phenomenon is called oncogene addiction and theoretically limited the growth-inhibitory effect of the inhibitor to the cancer cells with altered AKT signaling without affecting normal cells. Furthermore, combinatory inhibition of AKT1 and AKT2 is more effective in inducing apoptosis than inhibition of AKT1 or AKT2 alone and only combinatory inhibition of AKT1/2 sufficiently blocks AKT downstream signaling. AKT3 does not compromise the inhibition by the AKT1/2-inhibitor. The suppression of tumor growth through decreasing cyclin D levels and upregulation of p27 was accompanied by only a moderate and transient hyperglycemia in mice [12, 13, 182, 183]. The AKT1/2-inhibitor alone exerts only a slight apoptotic stimulus that can be maximized by combination with chemotherapeutics like camptothecin, γ-radiation or Herceptin treatment in vitro, proposing a sensitization for the anti-tumor treatments through the AKT1/2-inhibitor [13]. The AKT1/2-inhibitor is confronted with a higher resistance in TNBCs than in breast cancers of the luminal subtype. This could be explained by the higher dependency on AKT signaling in luminal breast cancer compared to the partly ERK-dependent TNBCs [184]. Knockdown of AKT3 sensitizes TNBC cells to a pan-AKT inhibition [151], whereas a E17K AKT1 mutation causes resistance to the AKT1/2-inhibitor [74]. Interestingly, treatment with the pan-AKT inhibitor AZD5363 in a clinical trial showed a higher efficacy when the tumor carries an E17K AKT1 mutation compared to tumors with wild type AKT1 [185]. Breast cancer cells treated with allosteric or ATP-competitive AKT inhibitors like MK2206 can develop a resistance against them through upregulation of AKT3, but not AKT1 or AKT2, via epigenetic changes [132]. Certain other isoform-specific inhibitors with promising efficacy in vitro and in vivo were developed in the last years, especially naphthyridine and naphthyridinone dual AKT1/2 inhibitors [186, 187]. As far as we know, further investigations to clarify the advantages of isoform-specific inhibitors in breast cancer and the efficacy and toxicity in clinical trials are still outstanding.

Combination of paclitaxel with an AKT1 shRNA synergistically inhibits tumor growth in vitro and in a mouse model. Accountable for this growth-inhibitory effect is an anti-proliferative effect via inhibition of Cyclin A, cyclin D1, cyclin D2, CDK2, CDK4 and PCNA as well as a pro-apoptotic effect via induction of Caspase 3 and BAD, inhibition of Bcl proteins and inhibition of tumor angiogenesis via inhibition of VEGF expression [188].

A clinically important characteristic of breast cancer cells is their response to radiation. Therefore, Toulany et al. reported a radiosensitization through DNA double-strand breaks of K-Ras mutated cells lacking AKT1 or AKT3, but not AKT2. This is due to a lack of the activating interaction between AKT1 or AKT3 with DNA-PKcs which is concerned in the repair of DNA double-strand breaks, as it was also reported by Baek et al. for BRCA1 and RAD51 [131]. As a consequence, AKT1 overexpression leads to a resistance to radiation [162].

## AKT isoform specificity in breast cancer: E17K AKT1 mutation and transforming ability

Park et al. revealed that AKT1 has no transforming ability in mammary epithelial cells, despite AKT in general transforms mammary epithelial cells [59]. Overexpression of AKT1 in mammary epithelium triggers the formation of benign lesions, but transformation to malignant lesions requires additional carcinogenic signals [119]. Contrary, another study detected a transforming ability of AKT1 in breast cancer through enhanced proliferation and suppressed apoptosis [143].

Carpten et al. first discovered a missense E17K mutation of AKT1 in breast cancer that causes a lysine substitution to glutamic acid at amino acid 17 due to a point mutation from G to A at nucleotide 49. This leads to a conformational change of AKT1 that exhibits a 100-fold higher affinity to PI(4,5)P_2_ and a 7-fold higher affinity to PI(3,4,5)P_3_ and therefore shows a constitutive membrane localization. Finally, the phosphorylation at T308 and therefore activation of AKT1 is enhanced in the E17K AKT1 mutation which shows transforming abilities in fibroblasts [71, 74, 189].

But Lauring et al. reported that knock in of the E17K AKT1 mutation in mammary epithelial cells is not able to transform the cells, indicated by a missing induction of colony formation, EGF-independent growth and a lack of altered mammosphere architecture. Although the mutation constitutively activates AKT1, activity of downstream proteins like Cyclin D1, pGSK and mTOR are not significantly altered by the mutation [190]. Comparison of the AKT1 E17K mutation and the mutation of PIK3CA displays enhanced AKT1 activity in the AKT1 mutation, but elevated activity of AKT1 and AKT2 in the PIK3CA mutated breast cancer cells. Notably, both mutations do not affect the downstream proteins mTOR, p70S6K, pS6, EIF4EBP1 and Cyclin D1, but the E17K AKT1 mutation enhances phosphorylation of FOXO1/3/4, PRAS40 and AS160, the latter one actually being an AKT2-specific substrate. The AKT1 mutation increases proliferation and tumor growth of breast cancer cells to an intermediate extent between wild type and PIK3CA mutated cells. While the PIK3CA mutation sensitizes cells to PI3K inhibitors as well as AKT inhibitors, AKT1 mutation lacks to sensitize cells to the PI3K inhibitor like LY294002, probably through the downstream position of the AKT1 mutation and therefore partly persisting AKT signaling upon treatment. Consistent to Lauring et al., the E17K AKT1 mutation only slightly sensitizes breast cancer cells to the pan-AKT inhibitor MK2206 [189–191].

E17K mutated AKT1 shows different effects in mammary epithelial or myoepithelial cells and in the corresponding transformed cells. In the non-transformed luminal epithelial cells, the mutation inhibits cell growth, migration and protein biosynthesis and increases cell survival, whereas in the transformed cells the mutation leads to suppressed cell growth and protein biosynthesis too but enhances cell survival and cell migration. In contrast, in the myoepithelial normal breast cancer cells growth, migration, survival and protein biosynthesis are reduced among mutated AKT1 and the transformed myoepithelial cancer cells with the E17K mutation exhibit a similar phenotype except of a missing effect on cell survival. Consistent with the findings in cell survival, the E17K AKT1 mutation decreases sensitivity of normal and transformed luminal cells to paclitaxel and etoposide, whereas in normal myoepithelial cells E17K increases sensitivity. There is no significant effect in the transformed counterpart. The partly promoting effect of the AKT1 mutation on migration conflicts with general findings in AKT1 knockdown studies that are listed above. In summary, the E17K mutation of AKT1 reduces cell growth and downregulates EGFR as well as it attenuates induction of p70S6K, indicating a lower protein biosynthesis. It follows that the E17K mutation decreases the DDX21 levels, a protein that is important for RNA processing e.g. splicing. Impaired protein biosynthesis and defective posttranscriptional modifications might account for the growth inhibitory effects and therefore lack of transforming abilities [189]. Furthermore, the E17K mutation of AKT1 was found to be still regulated by extracellular stimulation of the PI3K/AKT pathway e.g. by insulin stimulation [189, 192].

## AKT isoform specificity in breast cancer: expression, amplification and mutations

The investigation of AKT isoform expression and activation in human breast cancer probes and breast cancer cell lines detected expression of AKT1 and AKT2 in all breast cancer cell lineages with a higher abundance in the luminal breast cancer subtype. AKT3 expression was detected only in a subpopulation of breast cancer and its expression is correlated with a TNBC subtype. Besides, levels of pAKT1 are quite similar among the cell lines, whereas the phosphorylation of AKT2 and AKT3 varies in a wide range between the cells [178, 182, 193, 194]. Expression of AKT1 and AKT2 are not associated with each other in breast cancer lineages, indicating that they are independently regulated, but phosphorylation of AKT1 and AKT2 are correlating, indicating that the isoforms are both phosphorylated in a similar way [169]. Moreover, mRNA levels of AKT1 and AKT3 are correlated inversely, suggesting a compensatory role for each other [101]. The three AKT isoforms are localized at different subcellular compartments. In the study of Santi et al. AKT1 was found mainly in cytoplasm, AKT2 at the mitochondria and AKT3 in the nucleus. This subcellular localization is present in mammary epithelial cells and breast cancer cells, suggesting isoform localization within the cell is not critical for breast cancer development. Furthermore, the colocalization of AKT2 with mitochondria confirms its pivotal role in energy metabolisms and regulation of apoptosis [194]. Inconsistently, Spears et al. detected AKT1, pAKT1 and pAKT2 in the nucleus and in the cytoplasm. AKT2 was localized exclusively in the nucleus [195]. In contrast another study reported a localization of AKT1 in nucleus and cytoplasm, whereas expression of AKT2 and AKT3 was limited to the cytoplasm in ER-positive breast cancer. Cytoplasmic expression of AKT1 and AKT3, but not AKT2, is associated with cytoplasmic pAKT abundance [81]. TIS21 was shown to induce the translocation of AKT1 and pAKT1 to the nucleus, resulting in detection of AKT1 and pAKT1 in cytoplasm and nucleus [152], whereas Plant et al. reported a shift of AKT1 staining from nucleus to cytoplasm during breast cancer progression [196].

The frequency of AKT1 expression of breast cancer in general amounts to about 24%. But viewed critically, no AKT isoform expression was observed in corresponding normal breast tissue in this study [82]. A high AKT1 activation was observed in 19.9% of all breast cancer probes and 45% of human ductal breast cancer, the latter mainly due to high PI3K activity [195, 197]. High levels of pAKT1 are associated with a high grade and a high stage of the tumor, suggesting a pivotal role of AKT1 in tumor progression. Activated AKT1 was found most notably in cytoplasm and at the plasma membrane but not in the nucleus [197]. Poorly differentiated mammary tumors have low levels of AKT1 and AKT2, proposing a pivotal role of both isoforms in differentiation of breast tumors [198]. AKT1 expression was found to be lower in breast cancer than in corresponding normal mammary tissue, perhaps pointing to the anti-migratory effect of AKT1 [152]. In contrast, another study observed no difference of the expression of AKT1, AKT2 or AKT3 between breast cancer and normal mammary tissue [199].

Gene amplifications of AKT2 occur in 2.8% up to 4% of human breast carcinomas, although the number of breast cancer samples with amplified AKT2 is lower than in ovarian cancer [75, 82]. Additionally, high AKT2 kinase activity is displayed in 40% of breast cancer probes and is associated with late stage tumors, confirming a crucial role of AKT2 in tumor progression rather than in tumor initiation [179]. Conversely, Spears et al. detected high pAKT2 in only 17.4% of breast cancer [195]. Investigations of ER positive human breast cancer probes revealed AKT1 deletions in 4.8%, AKT1 amplifications in 1%, AKT2 deletions in 21.1%, interestingly no AKT2 amplifications, no deletions of AKT3, but AKT3 amplifications in 9.9% of investigated ER-positive human breast cancers [200].

In metastatic HER2-positive breast cancer a high AKT1 level was found in 12.2% of the samples and high AKT2 levels occurred in 35.1%. There was no correlation between the expression of AKT1 or AKT2 and ER or PR positive status in this study [201]. In contrast, the study of Carmona et al. reported no alterations in AKT2 among HER2-positive breast cancers, but they found alterations of AKT3 in 10% of HER2 breast cancer (mostly amplifications) and alterations of AKT1 in 2.5% (mostly E17K mutations) [202]. However, Bacus et al. reported an association of overexpression of AKT2, but not of AKT1, with a positive HER2 status and enhanced pan-AKT activation. Combined with the data that PI3K-inhibitors can sensitize HER2-positive breast cancer cells to hypoxic stress and that overexpression of HER2 leads to overexpression of AKT2, a HER2-mediated pro-survival signaling via AKT2 is suggestable [203].

As AKT1 is associated mainly with a positive ER status and its expression is lower in TNBC compared to the other subtypes, AKT2 expression was reported to correlate inversely with expression of ER [82, 119, 144]. A tissue-microarray of invasive breast cancer samples also revealed a positive association of AKT1 expression with ER and HER2 status. Furthermore, an inverse correlation between AKT1 expression and tumor stages as well as metastatic nodal status was detected [82, 204]. On the contrary, Sun et al. observed a positive correlation between increased AKT2 kinase activity and a positive ER-status, confirming the findings about increasing transcriptional activity of the ER by AKT2 [179]. Complementary AKT1 is associated with luminal B and HER2 subtypes, whereas AKT2 expression is associated with luminal A and luminal B subtypes [101].

Because AKT3 has a predominant effect in TNBC, the studies addressing expression, activation and correlations of AKT3 will be approached separately hereafter. Hu et al. reported that AKT3 expression can be found to a higher extent in breast cancer tissue than in the adjacent normal breast tissue [155]. But another study observed that AKT3 expression at the RNA level is not exclusively for human breast cancer probes and can also be found in normal breast tissue. The expression of AKT3 was not significantly associated with the hormone status in this study, although all investigated TNBCs expressed AKT3 [101, 199]. AKT3 expression in breast cancer is associated with an ER-negative receptor status in breast cancer cell lines and human samples, suggesting AKT3 contributes to aggressiveness in hormone receptor negative breast cancer [99]. According to the TCGA, 28% of breast cancers are AKT3 amplified [205]. Another study points out the importance of AKT3 in TNBC by reporting amplification of AKT3 in 14% of TNBC versus 3% of luminal breast cancer and upregulation of mRNA in 21% of TNBC versus 2% of luminal human breast cancer according to a TCGA analysis [151]. O’Hurley et al. disclosed significantly more AKT3 amplifications in TNBC with a frequency of 11% compared to 1% in ER-positive breast cancer, but also the amount of AKT3 deletions is even higher in TNBC with a frequency of 13% compared to 1% in ER-positive breast cancer. Finally, there is no significant difference in the expression of AKT3 in TNBC compared to ER-positive breast cancer, but a higher copy number of the AKT3 gene exists in TNBC [100]. Dividing AKT3 in its two different splice variants, the pro-tumorigenic AKT3 + S472 is highly expressed in TNBC, whereas the anti-tumorigenic AKT3-S472 is expressed to an lower extent [96].

This has been complemented by data indicating a higher AKT3 expression and activation in HER2-positive breast cancer, but not in ER-positive cells. Knockdown of AKT3 results in posttranscriptional downregulation of HER2/3 and the poor-prognosis marker FoxM1. Further on, ablation of AKT3 decreases phosphorylation of HER2/3 and finally enhances expression of ER via a decreased AKT3 dependent inactivating phosphorylation of Foxo3a. In addition, AKT3 mediates resistance to tamoxifen in HER2-positive breast cancer cells. Thus, AKT3 expression is higher in HER2-positive human breast cancer and TNBC than in ER-positive mammary tumors [130].

The detection of AKT3 expression in breast cancer-derived DTCs in the human bone marrow suggests a pivotal role of AKT3 in DTCs [206]. The role of AKT isoforms in CTCs was further outlined by two studies. Increased AKT2 levels in blood samples of metastatic or non-metastatic breast cancer patients serves as a marker for EMT and therefore predicts detection of CTCs. 62 to 70% of patients with CTCs showed detectable AKT2 in these studies [207, 208].

An own analysis of the Cosmic database revealed that AKT1 somatic mutations occur often and are one of the Top 20 mutated genes in breast cancer with a frequency of about 2.9%. The greatest part of the AKT1 mutations are missense E17K mutations, followed by L52R mutations. Mutations of AKT2 and AKT3 are rare events in breast cancer with a frequency of 0.4% each [209]. According to data from the TCGA AKT1 mutations emerge in about 2.4% of breast cancer, most of them in the luminal A subtype and none of them in basal like breast cancer [205].

The information about the frequency of the E17K AKT1 mutation differ in the literature from 0 to 8% and the mutation is found to be mutually exclusive [71, 74, 111, 190, 202, 210–218]. The occurrence is limited to hormone positive breast cancer and is only found in lobular and ductal breast cancer [71, 190, 214]. The E17K AKT1 mutation also occurs in DCIS, suggesting the mutation is an early event in breast cancer [210]. Troxell et al. observed a notably high frequency of the E17K AKT1 mutation in 54% in benign papillary neoplasm [219]. Stephens et al. detected a similar E17K mutation in AKT2 with a frequency of about 1% among breast cancer, although AKT2 is more frequently amplified in breast cancer as mentioned above [75, 218].

Some studies observed rare mutations of AKT1 like L52R, D32Y, K39 N, P42T, C77F, Q79K, E319G, L357P and P388T. L52R is the second leading mutation found in AKT1 in breast cancer [212, 218, 220]. But only the mutations L52R, C77F and Q79K were found to be relevant in human breast cancer and were shown to be constitutively localized at the plasma membrane. The mechanisms for the growth factor-independent membrane localization of these mutations is yet unknown. Furthermore, merely the mutations L52R, C77F and Q79K have transforming abilities, indicated by increased colony formation. The other non-transforming mutations could also be artefacts or passenger mutations and therefore should be interpreted carefully [220, 221]. López-Cortés et al. revealed further SNPs of AKT1 namely rs2494732 with a frequency of 14,3% and rs3803304 with a frequency of 7,7%. The first one was associated with a lower risk to develop breast cancer and the second one was associated with a higher risk of breast cancer among the population [212].

Additionally, in the study of Carmona et al. a mutation of AKT3, namely R247C, with a similar activating mechanism as the E17K mutation in AKT1 emerged in a breast cancer during trastuzumab treatment [202]. In about 3% of human breast cancer samples, preferably in TNBCs, Banerji et al. discovered a new balanced translocation in chromosome 1 resulting in a MAGI3-AKT3 fusion protein. This fusion protein combines the loss of function of PTEN (by MAGI3) and the activation of AKT3 and therefore shows high growth factor-independent AKT3 activation. Moreover, this newly described translocation of AKT3 predisposes to resistance against the pan-AKT inhibitor MK2206 in breast cancer and to malignant transformation in fibroblasts [213, 222].

Comparing chromosomal aberrations in primary tumors to corresponding metastases revealed the following values: In primary tumors AKT1 mutations occur in 2.8%, AKT2 amplifications in 2.8% and AKT3 amplifications in 5.6%. The corresponding metastases in turn show AKT1 mutations in 2.3%, AKT2 is amplified in 2.3% and AKT3 is amplified in 9.3%. In conclusion the frequency of AKT3 amplifications is increased in metastases compared to the primary tumors, but frequency of AKT1 mutations and AKT2 amplifications are similar in primary and metastatic tumors [223].

## AKT isoform specificity in breast cancer: overall survival, metastasis-free survival and treatment response

Consequently, the AKT isoforms have different effects on prognosis, therapy response and metastases formation in human breast cancers. According to an analysis of the TCGA, high AKT2 at the mRNA level, but not AKT1, is associated with a lower overall survival in 1105 cases of invasive breast cancer [127]. According to the study of Loi et al., ER+/HER- breast cancer samples with an E17K mutation of AKT1 are suspected to be associated with an improved prognosis [111]. High pAKT1 leads to enhanced cytoplasmic expression of Skp2 and this in turn is associated with large tumor size, high grade tumors, HER2 expression and an impaired disease free and overall survival in human breast cancer [224]. A high expression of AKT1 is associated with an improved overall survival [170]. While expression of AKT1 or AKT2 showed no correlation with prognosis in another study, a high level of pAKT1 is associated with lower overall survival and higher tumor size. High levels of pAKT2 are linked with poor overall survival only in ER-negative breast cancer. Surprisingly, pAKT2 can compensate and improve the poor prognosis in breast cancer with high pAKT1 [195]. By analyzing three datasets of breast cancer probes, Pérez-Tenorio et al. revealed a correlation of AKT1 expression with poor prognosis in the subgroup of ER-positive breast cancer, whereas AKT2 or AKT3 expression is associated with poor prognosis in breast cancer with ER-negative status [101]. A poor overall survival was found in ER-positive breast cancer with high copy numbers of the AKT3 gene [100].

In HER2-positive metastatic breast cancer that is treated with trastuzumab a high expression of AKT2, but not AKT1, in particular combined with a high level of pAKT T308, is associated with an improved overall survival and time to progression, but not with a disease free survival [201]. In accordance, high levels of AKT2, but not AKT1 or AKT3, in combination with low levels of pAKT S473 are associated with a good overall survival and disease-free survival in ER-positive breast cancer under adjuvant therapy with tamoxifen. Furthermore, this study revealed a crosstalk between the HER2-receptor and the ER-receptor via the PI3K/AKT axis mediating a tamoxifen resistance [81]. The study of van Agthoven et al. failed to detect any predictive values of AKT1 or AKT2 expression on response of breast cancer to tamoxifen [198]. In addition, resistance to tamoxifen treatment was found to be not associated with alterations in expression of any AKT isoform, but resistance to tamoxifen is associated with a higher amount of pAKT1 and therefore AKT1 kinase activity in an ER-positive breast cancer cell line [225]. On the other hand, AKT3 overexpression in an ER-positive breast cancer cell line causes resistance to tamoxifen [153].

The response to common chemotherapeutics in breast cancer is also determined in an isoform-specific manner but is only investigated in breast cancer cell lines. Phosphorylated AKT1 and partly high AKT1 levels are responsible for resistance of breast cancer cell lineages to paclitaxel, doxorubicin, gemcitabine, 5-fluorouracil, etoposide, camptothecin and tamoxifen through anti-apoptotic effects. The resistance to gemcitabine is in part explained by regulation of PDK1 acting upstream of AKT1. Therefore, knockdown of AKT1 sensitizes the cells to the chemotherapeutics by promotion of drug-induced apoptosis [142, 162, 226–229]. Overexpression of AKT1 sensitizes for mTOR treatment that in turn decreases IC50 values of doxorubicin, etoposide and tamoxifen, suggesting combination of mTOR treatment and chemotherapeutics in breast cancer with high AKT1 levels is considerable [228]. Taylor et al. observed that AKT1 can mediate resistance to chemotherapeutics and tamoxifen by cooperating with ERK activation [229]. MiR-17/20 increases apoptosis and sensitivity to doxorubicin and tamoxifen by increasing the p53 levels, at least partly through AKT1 [141]. Detection of AKT2 in blood samples as a predictor for presence of CTCs shows an impaired therapy response [207]. Though AKT2 also predicts existence of CTC in non-metastatic breast cancer patients, its detection is not associated with any clinicopathological parameter in this subgroup of breast cancer patients [208].

Low expression of AKT1 in combination with high levels of TSC2 is associated with diminished metastasis-free survival according to the anti-metastatic role of AKT1 in vitro and in vivo [129]. High levels of AKT2 or low levels of AKT1 together with high levels of the transcription factor Twist occur frequently in highly invasive human breast cancer with an EMT-phenotype [164, 170, 175]. A low ratio of AKT1 to AKT2 is frequently found in metastatic breast cancer compared to primary tumors and is associated with low miR200 and low E-cadherin levels, indicating an EMT phenotype. This points out an important role of the balance between the AKT isoforms in prognosis and metastasis of human breast cancer [163]. But in contrast, a high expression of AKT2, but not of AKT1, was found to be associated with longer metastasis free survival in ER-positive breast cancer without tamoxifen treatment. This positive effect on metastasis free survival was even stronger in the subpopulation with low EGFR levels [198]. Fohlin et al. confirmed the lower rate of distant recurrence in high AKT2 expressing ER-positive breast cancer cases, although AKT2 is higher expressed in ER-negative tumors. The prognostic prediction of AKT2 is even stronger in tumors with low AKT1 expression, whereas AKT1 expression was associated with PIK3CA mutations and had no prognostic value in this study [230]. The amount of pAKT1 was found to be associated with lower distant relapse free survival, whereas pAKT2 only shows the same association in ER-negative breast cancer [195]. Hohensee et al. reported an association between loss of PTEN, and therefore specifically higher AKT1 activity, with an impaired overall survival in brain metastases of breast cancer [171]. An elevated copy number of the AKT3 gene is negatively associated with recurrence free survival in TNBC [100].

## Discussion

### Summary

**Fig. 1:**
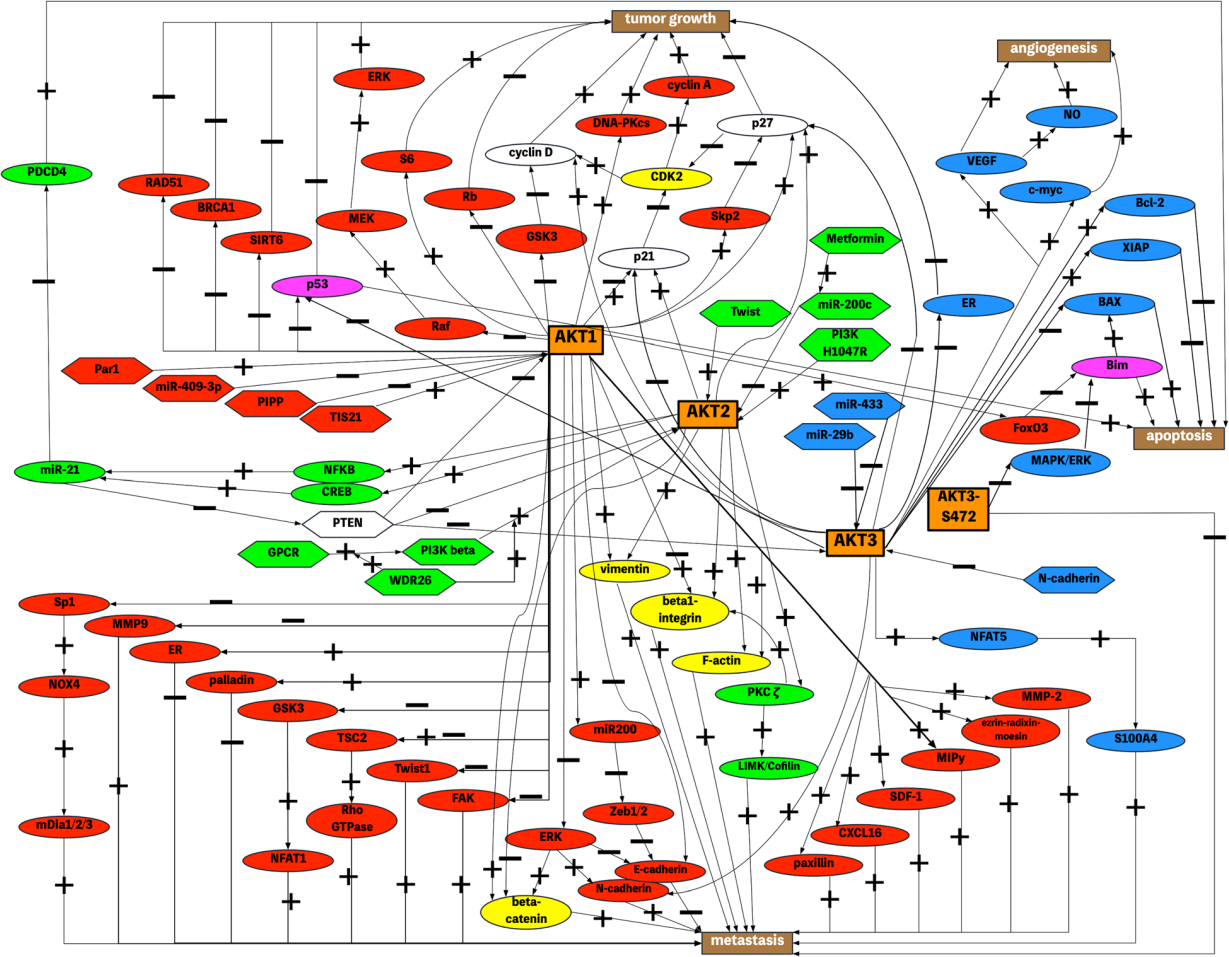
Isoform-specific AKT signaling in tumor growth, metastasis, apoptosis and angiogenesis of breast cancer. Figure 1 provides an overview of isoform-specific AKT signaling in the regulation of tumor growth, metastasis, apoptosis and angiogenesis in breast cancer. Orange rectangles show AKT isoforms and splice variants, brown rectangles represent cellular effects in breast cancer. Ellipses indicate downstream effectors of AKT isoforms, hexagons indicate upstream regulators of AKT isoforms. Red colored shapes represent upstream and downstream proteins of AKT1, green colored shapes represent upstream and downstream proteins of AKT2 and blue colored shapes represent upstream and downstream proteins of AKT3. Yellow shapes represent effectors of AKT1 and AKT2, magenta shapes represent effectors of AKT1 and AKT3 and white shapes present effectors or regulators of AKT1, AKT2 and AKT3. The position of the arrow head symbolizes the direction of interaction. A plus associated with lines represents an activating or upregulating interaction, a minus represents a suppressing or downregulating interaction

The data presented here point out the importance of the AKT isoforms in regulating the hallmarks of breast cancer like proliferation, apoptosis, migration, invasion and altered metabolism. Alterations in the PI3K/AKT signaling pathway occur frequently in breast cancer, supporting the importance of AKT as a potential approach for targeted therapy. Although, AKT1, AKT2 and AKT3 share a high homology, are activated and regulated by the same upstream mechanisms and share a wide range of substrates, the three isoforms exert non-redundant and partly opposing roles in breast cancer. First evidence for the non-redundancy in AKT isoforms was provided by observing AKT isoform-specific knockout mice. In the last 20 years several studies made the isoform-specific effects on breast cancer a subject of discussion. Besides the discovery of isoform-specific effects on breast cancer in vitro, in vivo and in human probes, a lot of considerable mechanisms were identified by which the AKT isoforms mediate their effects. The most important results were summarized in Table 1 and Fig. 1. A basic principle of AKT isoforms concluded from these data could be: One AKT isoform does not only exert one function but is responsible for several functions in the cell. In addition, more than one isoform regulates one cell function by using isoform-specific distinct pathways. Surprisingly, the studies showed partly contradictory and incompatible results by investigating the influence of the same isoform on the similar cellular process by using partly different methodical approaches and breast cancer cells with different genetic backgrounds. But even usage of equal approaches, the same cell lines and equal definitions sometimes produce inconsistent findings. Nevertheless, the isoform-specific effects on breast cancer can be summarized by determining the greatest consensus of the findings.

**Table 1:** shows essential AKT isoform-specific effects in breast cancer in vitro and in vivo classified by the revealing study and the three AKT isoforms AKT1, AKT2 and AKT3

Author	Ref.	AKT1	AKT2	AKT3
Hutchinson et al. 2004	[125]	tumor growth & proliferation ↑ (Rb, cyclin D1); metastasis ↓		
Dillon et al. 2009	[123]	tumor growth ↑; metastasis ↓ (ER)	tumor growth Ø; metastasis & invasion ↑	
Maroulakou et al. 2007	[124]	tumor growth & proliferation ↑ (Cyclin D1, Rb); apoptosis ↓; invasion ↓; metastasis ↑	tumor growth ↓ (Cyclin D1, Rb); metastasis ↑	tumor growth Ø metastasis Ø
Riggio et al. 2017	[127]	proliferation & tumor growth ↑ (Cyclin D1, S6); invasion & metastasis ↓ (integrin ß1, FAK, MMP9); migration Ø	proliferation ↓; tumor growth Ø; migration & invasion & metastasis ↑ (F-actin, vimentin)	
Liu et al. 2006	[129]	proliferation & tumor growth ↑; migration & invasion ↓ (TSC2, Rho)		
Grabinski et al. 2014	[130]	proliferation ↓	proliferation ↓	proliferation ↓
Toulany et al. 2017	[131]	proliferation & tumor growth ↑ (DNA-PKcs)	proliferation Ø; tumor growth ↓	proliferation & tumor growth ↑ (DNA-PKcs)
Park et al. 2001	[59]	colony formation Ø; invasion & metastasis ↑ (MMP-2)		
Stottrup et al. 2016	[132]	spheroid growth ↑	spheroid growth Ø	spheroid growth ↑; invasion ↑ (N-cadherin)
Chin et al. 2014	[133] [151]	proliferation ↑; spheroid formation ↑; migration ↓	proliferation ↑; spheroid formation ↑; maintaining spheroid architecture ↑; migration ↑	proliferation ↑; spheroid formation ↑; tumor growth ↑ (p27); spheroid growth ↑; migration ↓
Yang et al. 2011	[126]	proliferation ↓ (Raf/MEK/ERK); migration ↓	proliferation ↓ (p27, CDK2); migration ↓	
Ju et al. 2007	[136]	proliferation & tumor growth ↑ (p21, p27, Cyclin D1); migration & metastasis ↑ (TSC2, F-actin, MIPγ, SDF-1, CXCL-16, paxillin and ezrin-radixin-moesin); angiogenesis ↓		
Santi and Lee 2011	[128]	proliferation ↑	proliferation ↑ (CDK2, Cyclin D, p27); mitochondrial autophagy ↓ (PGC1, p70S6K)	proliferation ↑
Wang et al. 2008	[137]		proliferation ↑; migration & chemotaxis ↑ (PKCζ, LIMK/Cofilin, integrin ß1)	
Zhang et al. 2017	[138]		proliferation & tumor growth ↑; apoptosis ↓; migration & invasion ↑ (miR-200c)	
Polytarchou et al. 2011	[139]	tumor growth Ø	tumor growth ↑ (NF_K_B, CREB, miR-21, PTEN, PDCD4, Sprouty1)	
Ye et al. 2016	[140]		proliferation & tumor growth ↑; migration & invasion & chemotaxis ↑ (WDR26, PI3Kβ, Gβγ)	
Yu et al. 2014	[141]	apoptosis ↓ (p53, miR-17/20)		
Thirumurthi et al. 2014	[142]	tumor growth ↑ (SIRT6)		
Plo et al. 2008 Baek et al. 2018	[143] [121]	tumor growth & genomic instability (BRCA1, RAD51)		
Ooms et al. 2015	[144]	proliferation & tumor growth ↑; apoptosis ↓; migration & invasion & chemotaxis & metastasis ↓ (PIPP)		
Zhang et al. 2016	[145]	proliferation & tumor growth ↑ migration & invasion ↓ (miR-409-3p)		
Yang et al. 2009	[146]	apoptosis ↓; tumor growth ↑; invasion & metastasis ↑ (Par1)		
Watson and Moorehead 2013	[147]	proliferation & tumor growth ↑; metastasis Ø	proliferation & tumor growth ↑; metastasis Ø	
Irie et al. 2005	[148]	migration ↓ (ERK, E-cadherin, N-cadherin); proliferation ↑	migration ↑ (vimentin); proliferation ↑	
Gargini et al. 2015	[149]	proliferation ↑; apoptosis ↓ (FoxO3, Bim); mammosphere growth ↓ (Bim, E cadherin, vimentin, ß-catenin, integrin β1)	proliferation ↑; mammosphere growth ↓	
Hu et al. 2018	[155]			proliferation ↑; apoptosis ↓ (miR-433, Bcl-2, BAX)
Li et al. 2017	[156]			proliferation & tumor growth ↑ apoptosis ↓ (p53, p21, p27, CyclinD1, Bcl2, XIAP); migration & invasion ↑ (miR-29b); angiogenesis ↑ (VEGF, c-myc)
Suyama et al. 2018	[96]			AKT3-S472: proliferation & tumor growth ↓; apoptosis ↑ (Bim, MAPK/ERK, BAX); metastasis ↓
Lehman et al. 2012	[157]	invasion ↑ (RhoC GTPase)	invasion ↑	proliferation ↑; apoptosis ↓
Li et al. 2018	[113]	invasion ↓ (ERK, ß-catenin)		
Chung et al 2013	[154]			proliferation Ø; migration & invasion ↓ (N-cadherin)
Chin and Toker 2010, 2014	[158] [159] [160]	migration ↓ (palladin)		
Yoeli-Lerner et al. 2009, 2005	[112] [161]	migration ↓ (GSK3, HDM2, NFAT1)		
Choi et al. 2016	[152]	migration & invasion ↓ (TIS21, Sp1, NOX4, mDia1/2/3)	migration & invasion Ø	
Iliopoulos et al. 2009	[163]	TGF-β stimulated migration & metastasis ↓ (miR-200, Zeb1/2, E-cadherin); spheroid formation ↓	migration & metastasis Ø; spheroid formation Ø	
Cheng et al. 2007, 2008	[164] [168]		migration & invasion ↑ (Twist)	
Leal-Orta et al. 2018	[165]		migration & invasion ↑	
Marcial-Medina et al. 2019	[166]		migration ↑	
Arboleda et al. 2003	[167]	invasion Ø	invasion & metastasis ↑ (integrin β1); post invasion survival ↑	invasion Ø
Li et al. 2016	[170]	migration & invasion & metastasis ↓ (Twist1)		
Hohensee et al 2016	[171]	migration & invasion ↑; brain metastasis ↑		
Grottke et al. 2016	[150]	proliferation Ø; migration & chemotaxis Ø	proliferation Ø; migration & chemotaxis Ø	proliferation Ø; migration & chemotaxis & metastasis ↓ (S100A4, NFAT5)

Symbols have the following meanings: ↑ = increased, ↓ = decreased, Ø = no effect. Affected proteins and pathways are mentioned in the brackets

First of all, AKT1 turned out to be the predominant isoform for initiation of involution in the normal mammary gland and further influences the normal mammary gland formation and lactation. With regard to breast cancer, AKT1 plays a crucial role for proliferation and tumor growth in vivo by regulating the cell cycle and attenuates the influence of cell cycle inhibitors. Furthermore, apoptosis is inhibited by AKT1 fostering the induction of tumor growth. On the downside, AKT1 blocks migration and invasion in vitro, e.g. by inhibiting EMT or cytoskeleton reorganization, and suppresses metastasis in vivo. The actin-bundling protein palladin was the first isoform-specific substrate discovered and it is involved in motility of the cells, mediating the anti-migratory function of AKT1. Under some conditions, AKT2 is suggested to have an inhibitory effect on proliferation and tumor growth, however the role of AKT2 in cell cycle regulation and apoptosis is not as clear as the function of AKT1. Conversely, AKT2 clearly promotes migration and invasion e.g. through induction of an invasive phenotype and integrin-mediated ECM interaction. Moreover, AKT2 is the critical isoform mediating metastasis of breast cancer. Roughly summarized, AKT1 is important for breast cancer initiation and growth of the primary tumor, whereas AKT2 plays a pivotal role in progression of breast cancer by formation of metastases. AKT3 was found to play an important role in ER-negative breast cancer and TNBC. Although the effect of AKT3 depends to a large extent on the cells used for investigations, it seems like AKT3 rather has anti-metastatic, pro-proliferative and pro-oncogenic effects. When interpreting the findings concerning the functions of AKT3, we ought to keep in mind that most studies did not differentiate the both splicing variants of AKT3. They are known to exert distinct functions to some extent. However, the knowledge about the role of AKT3 in breast cancer is sparse and should be addressed by further investigations. Another notable mechanism by which AKT isoforms are not only regulated, but that also regulates their downstream effects are microRNAs which target proteins and therefore downregulate their abundance. Additionally, AKT1 and AKT3 are involved in regulating angiogenesis. Moreover, the studies found out that AKT1 and AKT2 regulate expression and transcriptional activity of the ERα in an isoform-specific manner.

Not least because of the missense E17K mutation of AKT1 that leads to a constitutive membrane localization and activation, AKT1 is the only isoform that showed partly transforming abilities. But the E17K mutation of AKT1 showed different results and therefore the clinical relevance is not clear at all. Studies investigating expression, amplification and mutation of AKT isoforms as well as their influence on prognosis and treatment response also show inconsistent findings. The results concerning the role of AKT isoforms on overall survival, treatment response and association with metastasis are summarized in Table 2. Whilst the most frequent alteration of AKT1 are mutations like the E17K mutation, AKT2 and AKT3 show a high frequency of amplifications. Likewise, a balanced translocation of AKT3 was found resulting in an MAGI3-AKT3 fusion protein. The expression and kinase activity at the protein level mainly depend on the subtype of breast cancer e.g. AKT3 is highly expressed in TNBC, whereas expression of AKT1 correlates with positive ER status. With a view to clinical relevance, AKT1, especially pAKT1, is associated with an impaired survival, whereas tumors with a high AKT2 expression respond better to tamoxifen and high levels of pAKT1 mediate resistance to common chemotherapeutics. As a logical consequence, AKT isoform-specific inhibitors were developed and tested. Although an AKT1/2-inhibitor showed good efficacy in vitro and in the mouse model, clinical investigations and establishment of AKT isoform-specific inhibitors are fragmentary and require more effort in the future. Since the AKT isoforms were shown to have different functions depending on stage and cell context, further investigations should focus on distinct processes in the progression of breast cancer. For instance, the specific role of the AKT isoforms in bone metastasis is a worthwhile issue, because bone metastasis is a frequent event in breast cancer and is associated with poor prognosis and lacking therapy [3].

**Table 2:** Impact of the AKT isoforms on survival, therapy response and metastasis. Shows the impact of AKT isoform expression, phosphorylation and mutation on clinical parameters, namely overall survival, therapy response and metastasis. For more detailed information about the used predictors for therapy response see the corresponding section above. The effects are classified by the revealing studies. If the effect is restricted to a subtype of breast cancer, this is shown in the brackets. Also, additional information is mentioned in the brackets

author	Ref.	effect
Riggio et al. 2017	[127]	high AKT2 ➔ reduced overall survival
Liu et al. 2012	[224]	high pAKT1 ➔ reduced overall and disease-free survival (association with Skp2 expression)
Li et al. 2016	[170]	high AKT1 ➔ improved overall survival low AKT1 and high Twist ➔ association with EMT high AKT3 ➔ association with EMT
Spears et al. 2012	[195]	high pAKT1 ➔ reduced overall survival and reduced metastasis-free survival high pAKT2 ➔ reduced overall survival and reduced metastasis-free survival (ER-)
Perez-Tenorio et al. 2014	[101]	high AKT1 ➔ poor prognosis (ER+) high AKT2 or AKT3 ➔ poor prognosis (ER-)
O’Hurley et al. 2014	[100]	AKT3 amplification ➔ recurrence-free survival (ER+) and reduced metastasis-free survival (TNBC)
Grell et al. 2012	[201]	high AKT2 ➔ improved response to trastuzumab (HER2+, metastatic)
Kirkegaard et al. 2005	[81]	high AKT2 ➔ improved response to tamoxifen (ER+)
Jordan et al. 2004	[225]	high pAKT1 ➔ reduced response to tamoxifen (ER+ cell line)
Faridi et al. 2003	[153]	high AKT3 ➔ reduced response to tamoxifen (ER+ cell line)
Knuefermann et al. 2003	[227]	high pAKT1 ➔ reduced response to paclitaxel, doxorubicin, 5-fluorouracil, etoposide, camptothecin (ER+ cell line)
Liang et al. 2006	[226]	high AKT1 and pAKT1 ➔ reduced response to paclitaxel, doxorubicin, gemcitabine (cell lines)
Sokolosky et al. 2011	[228]	high activated AKT1 ➔ reduced response to doxorubicin, etoposide, tamoxifen & improved response to mTOR inhibitor rapamycin (ER+ cell line)
Steelman et al. 2011	[162]	high activated AKT1 ➔ reduced response to doxorubicin and tamoxifen (ER+ cell line)
Taylor et al. 2011	[229]	high activated AKT1 in combination with ERK activation ➔ reduced response to doxorubicin and tamoxifen (ER+ cell line)
Thirumurthi et al. 2014	[142]	destabilization of SIRT6 by AKT1 ➔ reduced response to tamoxifen (HER2+ cell line)
Aktas et al. 2009	[207]	high AKT2 in blood as predictor for presence of CTCs ➔ reduced therapy response in general (metastatic breast cancer)
Liu et al. 2006	[129]	low AKT1 ➔ reduced metastasis-free survival (combinatory with low TSC2)
Cheng et al. 2007	[164]	high AKT2 and high Twist ➔ association with late stage and invasiveness of tumor
Iliopoulos et al. 2009	[163]	low AKT1/AKT2 ratio ➔ increased metastasis
van Agthoven et al. 2009	[198]	high AKT2 ➔ improved metastasis-free survival (ER+)
Hohensee et al. 2017	[171]	high AKT1 activity through loss of PTEN ➔ reduced overall survival in brain metastasized breast cancer

### Differences among the findings

Multiple mechanisms can cause the differences between the studies and will be discussed hereafter. Isoform-specific effects in cancer depend not only significantly on the cancer entity, but also on subtype and even on different cell lines of the same tumor entity. In contrast to the general findings in breast cancer, AKT1 knockdown in mouse embryo fibroblasts decreases migration and invasion, whereas AKT2 has an anti-migratory and anti-invasive effect. These findings can be explained by an inactivating impact of AKT2 and an activating impact of AKT1 on the pro-migratory Rac/Pak1-signaling which alters the actin cytoskeleton in mouse embryo fibroblasts [231]. In other gynecological tumors the AKT isoforms display effects which differ from that in breast cancer. Ovarian tumor cells with an AKT1 knockdown show impaired tumor progression and metastasis, whereas an AKT2 knockdown leads to increased tumor progression and metastases formation. The effect of AKT3 on the ovarian tumor cells consists in a moderate acceleration of tumor progression and metastasis. Interestingly, also knockdown of the corresponding isoforms in the mouse followed by the injection of unaltered ovarian cancer cells show the same effect on tumor progression, suggesting a crucial role of the tumor microenvironment on ovarian tumor progression and metastasis. The opposing effect of AKT1 on metastasis between ovarian cancer and breast cancer may be due to the different ways of metastasis: breast cancer metastases are vascular metastases, whereas ovarian cancer cells metastasize directly to the peritoneal cavity. The reason why AKT2 deficient mice facilitate tumor progression might be a hyperglycemic setting and therefore a heightened metabolism in the tumor cells due to the regulation of GLUT1 by AKT2 [232]. Furthermore, the well-known AKT substrate GSK3 is responsible for metabolism, as it was shown in renal caner [233, 234]. In combination with the fact that GSK3 is regulated by AKT1 in breast cancer regulating the migration, an isoform specific modification of metabolism as a hallmark of cancer can also be assumed in breast cancer [112, 161]. This hypothesis is supported by the findings of Gonzalez et al., showing that translocation of the glucose transporter GLUT4 due to insulin stimulation is an AKT2 specific effect in adipocytes [192, 235]. Lim et al. recently found out that the actin-capping protein Tropomodulin 3 is an AKT2 specific substrate mediating the translocation of GLUT4 to the membrane and therefore glucose uptake into the cell [236]. Further evidence on this issue represents the diabetes-like phenotype in mice lacking AKT2 [103, 104]. However, to our knowledge there is no publication so far dealing with the AKT isoform-specific effects on metabolisms particularly in breast cancer and therefore this hypothesis should be a topic of further studies.

Another study revealed that AKT3 induces cancer progression and growth in a subpopulation of ovarian cancer by mediating the G2-M-transition and therefore an increased cell proliferation. In opposition to breast cancer cells, ovarian cancer cells as well as non-transformed ovarian cells display a higher expression and activation of AKT3 compared to the other isoforms, suggesting AKT3 is more important in ovarian cancer than in breast cancer [237]. In contrast to the distinct functions of AKT1 and AKT2 in breast cancer, both isoforms exhibit a decreasing effect on migration, invasion and focal adhesion by inhibiting the activity of β1-integrin in prostate cancer cells which are also highly hormone-dependent like breast cancer cells. AKT1 mediates this effect through a negative regulation of receptor tyrosine kinases like EGFR, whereas AKT2 is a suppressor of the pro-migratory miR-200 family like in breast cancer cells [238].

Furthermore, the summarized data presented here suggest that isoform-specific effects depend on the ER and HER2 status of breast cancer cells as well as on different cell lines of the same molecular subtype. But the mechanism behind this phenomenon remains mostly unrevealed. Whilst high pAKT1 is associated with a low overall survival in breast cancer independent of the hormone status, pAKT2 functions as a predictor for poor prognosis only in ER-negative breast cancer [195]. In the study of Grottke et al. AKT3 has an anti-migratory effect in a TNBC cell line, but in another TNBC cell line used by another study AKT3 shows a pro-migratory effect [132, 150]. Moreover, Yang et al. revealed an anti-proliferative effect of AKT2 in a TNBC cell line, whereas Santi et al. observed the same phenotype by using a knockdown of AKT2 in the same TNBC cells [126, 128].

The difference in the findings of Yang et al. and Santi et al. using the same TNBC cell line might be explained by the difference in the methodical approach, namely an AKT2 overexpression in the study of Yang et al. and an AKT2 knockdown used by Santi et al. It could be speculated that an overexpression can cause a negative feedback regulation or inhibitory control mechanisms and therefore modify the isoform-specific effects [126, 128]. Another important point concerning methodical approaches is the usage of myr-AKT-isoform overexpression in which the AKT isoform is constitutively bound at the membrane. As a result, the possible role of the isoform-specific subcellular localization as a mechanism behind the isoform-specific effects might be abrogated. Particularly, the findings of Gonzalez et al. suggest a localization of distinct isoforms at the plasma membrane as a critical mechanism for isoform specificity, allowing to doubt the appropriateness of using myr-AKT-isoform overexpression [192]. These considerations are expanded by the fact that Sun et al. found a translocation of activated AKT1 to the nucleus in exogenous AKT1 overexpressing cells in vitro, but this does not occur in human specimen [197]. Also, the cell culture conditions have an explicit impact on the effect of AKT isoform-specific knockdown in regulating cell signaling. For instance, Gargini et al. observed a differential effect of AKT1 on expression pattern of epithelial and mesenchymal cells under adherent cell culture conditions with serum-rich medium compared to mammosphere culture conditions with growth factor containing medium [149].

Besides molecular alterations, the differential regulation of effector proteins by different pathways and the predominance of distinct pathways in different cell lines might account for differences among the studies. Thus, studies in the future should consider the dependency on AKT signaling of the investigated cells and a possible crosstalk with other pathways. Another possible explanation for the discrepancies might be a dose-dependency of AKT isoform-specific effects. For instance, in mammary gland involution a particular level of AKT2 is necessary for involution. Hence, increasing but also decreasing the AKT2 levels result in a delay of involution [123]. The disparity between effects on invasiveness in vitro and metastasis in vivo by the AKT isoforms, most notably AKT1, might be due to the complexity of the metastatic cascade in vivo. Hence, the outgrowth of tumors at the metastatic site of AKT1 knockdown cells might be impaired despite an enhanced invasion, because paracrine and autocrine mechanisms might influence the processes in vivo and interactions between tumor cells and the tumor surrounding stroma plays a pivotal role in tumorigenesis. Investigations in vitro should be completed by investigations in a mouse model to consider the possible influencing mechanisms in vivo [123, 124]. Data about the role of AKT isoforms in the tumor-associated stroma and therefore in cell-cell-interactions and paracrine and autocrine stimuli are sparse and need to be further elucidated.

It is important to note that differences between isoform-specific functions in mice might depend on the kind of mouse model used. This could be the reason why Maroulakou et al. revealed no anti-metastatic effect through ablation of AKT1 in the whole MMTV-PyMT mice due to a germline knockout, whereas Dillon et al. observed an anti-metastatic effect of AKT1 in MMTV-PyMT mice in which AKT1 was activated specifically in the mammary tissue. This contributes to the hypothesis of a relevant crosstalk between tumor cells and stromal cells in the process of metastasis [123, 124]. Another possibility for the lower amount of metastases in the studies of Ju et al. and Maroulakou et al. might be the impaired primary tumor growth in AKT1 knockout mice and therefore a lower amount of primary tumor cells that can form secondary metastases [124, 136, 170]. The discrepancies between the results of Maroulakou et al. and Watson and Moorehead can be explained by the usage of the different oncogenic drivers, namely IGF-1 and HER2. As a result, activation of the AKT pathway and other pathways as well as representation of different molecular breast cancer subtypes differ in both studies. Furthermore, both laboratories used mice with different genetic backgrounds. In addition, Watson and Moorehead collected evidence for compensating signaling pathways in AKT isoform knockdowns, as they observed an activation of ERK and other AKT isoforms in the AKT2 knockdown cells [124, 147]. Limitations of AKT isoform-specific investigations are attributable to the disparities among species so that even the transferability of findings in the mouse models should be interpreted carefully. For instance, investigations of the protein Par-4/PAWR in rats identified its direct targeting of AKT. But studies on human cells and in mice revealed that the suggested site in AKT is not conserved among humans or mice [45].

Studies investigating the prognosis, metastasis and treatment response in the context of AKT isoform-specific effects also show inconsistent results. This might be explained by different study populations with distinct genetic backgrounds, different technical approaches of AKT isoform detection and variable definitions of expression thresholds [127]. Moreover, when interpreting the influence of AKT isoforms on prognosis and therapy response the difference between the detection of AKT protein expression and the measurement of phosphorylated AKT or kinase activity should be noted. For example, differences between the findings of Kirkegaard et al. and van Agthoven et al. might stem from the lack of detection of the more important phosphorylated form of AKT2 in the study of van Agthoven et al. compared to the study of Kirkegaard et al. Besides, the effect of pAKT2 in the study of Kirkegaard et al. was independent of tamoxifen treatment and therefore perhaps could not be reproduced by van Agthoven et al. by focusing on the response to tamoxifen treatment [81, 198].

The isoform-specific effects can differ in the distinct stages of the breast cancer disease, e.g. AKT1 could promote tumor initiation and growth of the primary tumor, whereas AKT2 preferably promotes tumor progression and metastasis [123, 127]. The dichotomy of AKT1 in positively regulating proliferation and negatively regulating migration also suggests a stadium-dependent role of the AKT isoforms in breast cancer and was reviewed by Toker and Yoeli-Lerner in 2006 [239]. This tumor stage-dependent effect of AKT1 in breast cancer was first reported by Hutchinson et al. in 2004 by observing a decrease in tumor growth by suppressing AKT1 in a breast cancer mouse model, whereas the metastasis is increased [125]. As a consequence, AKT1 is supposed to play a crucial role in the initiation and progression of the primary tumor. Various studies confirmed this pro-proliferative and tumor initiating effect in vitro and in vivo and identified several downstream mechanisms e.g. cyclin D1 and Rb [125, 127] or p21 and p27 [136]. Because of the anti-metastatic effect of AKT1 in breast cancer, a loss of AKT1 expression or activity at least in a subpopulation of the primary tumor could be a possible mechanism for the initiation and development of metastases. Ablation of AKT1 promotes migration and invasion as key processes of the metastatic cascade for example by regulating integrin β1 and MMP9 [127], TSC2 [129], the AKT1-specific substrate palladin [159] and by promoting EMT [148]. The hypothesis about the dichotomy gained further confirmation from data of clinical breast cancer probes reporting an association of low AKT1 with a reduced metastasis-free survival [129]. However, it has to be taken into account that some studies could not reproduce this dichotomy by observing a pro-proliferative and pro-metastatic role of AKT1 [146] or a suppression of both processes [126]. Thus, studies that will be performed in the future should differentially evaluate the AKT isoform-specific effects in the various stages of the disease. Interestingly, the dichotomic role of AKT1 is not limited to breast cancer. Gao et al. discovered the pro-proliferative and anti-metastatic effect of AKT1 in prostate cancer. Ablation of AKT1 decreases prostate tumor growth in vivo but enhances formation of lung metastases. Moreover, the promotion of metastasis by AKT1 knockdown is mediated in a similar way compared to breast cancer. Knockdown of AKT1 in the prostate cancer cells promotes EMT as it was observed by Irie et al. in breast cancer [148, 240]. Surprisingly, AKT1 knockdown in prostate cancer cells resulted in a decreased β-catenin level [240], whereas the pro-metastatic effect of AKT1 in breast cancer was associated with a nuclear accumulation of β-catenin [113]. Alwhaibi et al. reported an activation of the FoxO3a-Nodal pathway in AKT1 knockdown prostate cancer cells leading to the pro-metastatic EMT of the cells [241]. Non-small cell lung cancer cells with KRAS or EGFR mutations and colorectal carcinoma cells also show the dichotomy of AKT1 during tumor progression and metastasis [242–244]. The anti-migratory and anti-invasive effect of AKT1 in colorectal carcinoma cells is mediated by a decrease in MMP9 expression, as it was also observed in breast cancer cells [127, 244]. It should be noted that other studies reported a pro-metastatic effect of AKT1 in prostate, lung and colorectal cancer denying the dichotomic role of AKT1 in these cancer entities [245–247]. In contrast to the findings on AKT1 in breast cancer and the cancer entities mentioned above, other subtypes of cancer do not display the tumor stage-dependent function of AKT1. For instance, in ovarian and hepatocellular cancer AKT1 induces proliferation and tumor growth as well as migration and metastasis [232, 244, 248]. In hepatocellular cancer expression of MMP9 is upregulated by AKT1 and thus controlled in the opposite direction compared to breast and colorectal cancer [127, 244]. This might explain the absent dichotomy of AKT1 in these cancer cells. Again, these considerations illustrate the tumor entity-dependent roles of AKT isoforms as a possible explanation for differences in AKT isoform-specific signaling among studies.

In contrast to the non-transforming abilities of AKT1 and AKT2 in the studies with MMTV-mice, a study using myrAKT isoforms in chicken embryo fibroblasts reported a transforming effect of all AKT isoforms in a non-isoform-specific manner [249]. This discrepancy in transforming abilities and the disparities between Carpten et al. and Beaver et al. on the one hand reporting an oncogenic potential of the E17K AKT1 mutation and Lauring et al. and Salhia et al. on the other hand denying a transforming ability might be explained by differences in the used methods. For example, transgenic overexpression was used by Carpten et al., whereas Lauring et al. used a knock in model. The different susceptibility to transforming stimuli and the genetic background of the different cells might also be an explanation. Another important point is the relatively higher affinity of E17K mutated AKT1 to PI(4,5)P_2_ than to PI(3,4,5)P_3_ and therefore perhaps an anti-oncogenic effect by binding to PI(4,5)P_2_ despite the constitutive membrane localization [74, 189–191]. Drawing a conclusion regarding the data about transforming abilities, mutations or overexpression of AKT isoforms can require further alterations in other pathways for transformation of mammary cells. Further studies should address these differences among the studies on a meta level, as this could provide other perspectives on the AKT isoform-specific functions.

### Mechanisms of isoform specificity

The possible mechanisms behind the isoform specificity in breast cancer remains mainly unrevealed. Several hypotheses exist to explain the isoform specificity of AKT and are discussed below [230, 235, 250–253]. Firstly, the isoforms of AKT are expressed in different amounts in the varying tissues, like the mainly restricted expression of AKT3 in neuronal tissue. This distinct expression and therefore tissue-specific importance of AKT isoforms might cause the cell-specific effect of the individual isoforms [94].

Secondly, the distinct isoforms are differently distributed in cell compartments as shown by Santi et al. and therefore interact with distinct substrates and adapter proteins [194]. For instance, insulin stimulation in adipocytes specifically translocates AKT2, but not AKT1, to the plasma membrane and therefore causes the AKT2 mediated GLUT4 translocation. The translocation of GLUT4 is mediated by an AKT2 specific phosphorylation of AS160 that is dependent on the specific localization of AKT2 to the plasma membrane [192]. In this study they were not able to identify a specific domain of AKT that mediates the isoform specific membrane localization. But another study reported that only the linker-region of AKT1 is responsible for specific translocation of this isoform to membrane ruffles upon PDGF stimulation in mouse fibroblasts [254]. Likewise, AKT2 is preferably localized at regions with cell-matrix contact of migrating cells, suggesting its colocalization with proteins that are responsible for motility like β-integrins [167].

Thirdly, the extracellular stimuli might activate their specific isoform pattern, probably through different amplitude or timing of the PI3K activity or PHLPP activity. Distinct isoforms of upstream proteins can regulate distinct AKT isoforms, hence the isoforms of PI3K are proposed to exert isoform-specific activation of AKT [253]. Confirming this Brognard et al. revealed that PHLPP1 specifically inactivates AKT2 and therefore downstream proteins like GSK3 and HDM2, whereas PHLPP2 dephosphorylates AKT3 and therefore specifically regulates p27 [39].

Fourthly, there might be an amino acid-dependent substrate specificity of the AKT isoforms, e.g. the AKT1-specific substrate palladin which specifically interacts with the linker region of AKT1. The linker regions of the AKT isoforms show the lowest homology among the isoforms. This suggests a possible isoform specificity that is determined by the differences in amino acid structure despite the high homology. Interestingly, different isoforms can be involved in different regulation mechanisms of the same protein, since AKT1 specifically regulates the phosphorylation and AKT2 regulates the protein levels of palladin [158–160].

Fifthly, some proteins interact specifically with an AKT isoform and influence its activity. AKT1 and AKT2 both interact with all TCL1 family members like TCL1, MTCP1, and TCL1b in leukemia cells and as consequence the activity of both isoforms is increased in an unspecific manner. In contrast AKT3 specifically interacts with TCL1, but not with MTCP1 or TCL1b and therefore specifically gets activated by TCL1. The isoform-specific interaction is mediated by differences in the PH-domain [12, 255]. Walz et al. reported a specific interaction between endosomal protein WDFY2 and AKT2, but not AKT1. Knockdown of WDFY2 results in decreased AKT2 levels and thus specific reduction of insulin-mediated AKT signaling [256]. CK2 phosphorylates AKT1 at Ser129 in the linker region leading to enhanced activation of AKT1 but does not phosphorylate the corresponding region in AKT2 in vivo. Phosphorylated AKT1 at S129 in turn promotes phosphorylation of palladin [257].

Sixthly, posttranslational isoform-specific modifications could play an important role. For instance, PDGF-mediated generation of reactive oxygen species results in specific oxidation of Cys124 of AKT2 leading to its inhibition [258].

Seventhly, microRNAs can regulate AKT levels and activity in an isoform-specific manner, as shown e.g. by Iliopoulos et al. (see sections above) [163]. But until now the precise mechanisms behind isoform specificity in breast cancer is still unclear and needs to be further elucidated.

### Clinical implications

Since the importance of the AKT signaling in breast cancer and other cancer entities is common knowledge, several laboratories made an effort to investigate the efficacy of AKT inhibitors in breast cancer. Another theoretical reason for using AKT as a therapeutical approach is the convergence of multiple upstream signaling in AKT [111]. Pan-AKT inhibitors like MK2206, GSK2141795 and AZD5363 are currently under clinical investigations, but no AKT isoform-specific inhibitor is used in clinical trials at the moment. The AKT1/2-inhibitor discovered by Barnett et al. showed toxicity in former clinical trials, despite the good results in preclinical trials [18, 101]. Additionally, pan-AKT inhibition with low dose MK2206 treatment can increase metastasis, probably by predominantly inhibiting AKT1, making an isoform-specific inhibition of AKT suggestable and an important content of further studies [113]. Isoform-specific inhibition of AKT1 and AKT2 showed an even higher effect than treatment with the mTOR inhibitor rapamycin in vitro [13]. AKT isoform-specific inhibitors could also be used to overcome resistance to chemotherapeutics or other targeted therapies that is driven by AKT activation. Despite the high homology in amino acid sequence among the AKT isoforms, there are certain regions, especially the linker region that are suitable for development of AKT isoform-specific inhibitors. By combination of this knowledge with modern drug analysis and development tools as it was shown by Akhtar and Jabeen, further AKT isoform-specific inhibitors can be developed with a more favorable drug safety [259].

But according to the current knowledge about isoform-specific effects in breast cancer, an isoform-specific inhibition of any isoform alone is not advisable before further investigations are made, because of the dichotomy of the isoforms. One possibility is to combine AKT isoform-specific inhibition with inhibition of downstream effectors that mediate the unfavorable effects of isoform inhibition. Hence, investigation of AKT isoform-specific substrates and downstream signaling makes further investigations necessary. Another option that could be considered is to inhibit isoform-specific downstream proteins of AKT (e.g. palladin). This might decrease side effects of the pan-AKT inhibitors e.g. a varied glucose metabolism, avoid unwanted effects emerging through dichotomy of the isoforms and represent a good alternative to AKT isoform-specific inhibitors [160]. For this approach it is also important to gain further understanding of the downstream signaling of the AKT isoforms to develop effective targeted treatment.

Besides this well-known clinical implication of isoform-specific AKT inhibition in breast cancer, there are some further clinical implications that can be reasoned from these studies. The knowledge of the different effects of the AKT isoforms in cancer progression and metastasis is not only important for an effective isoform-targeted therapy but probably also for usage as prognostic markers. Individual testing of AKT isoform expression and activation as molecular predictors for outcome and therapy response in patients could be implemented besides the evaluation of the HER2 and ER/PR status, e.g. by the newly described nanofluidic immunoassay [193]. For this purpose, further clinical studies addressing the role of AKT isoforms on therapy response, overall survival and distant relapse free survival are needed. It is conceivable to use drug testing, e.g. for AKT isoform-specific inhibitors or inhibitors of distinct downstream substrates, by using 3D spheroid cultures of breast cancer cells derived from patients. Several studies point out an existing difference between 2D culture and 3D spheroid growth experiments, suggesting spheroid testing is more appropriate to simulate the in vivo processes [133]. These considerations are approaches for a personalized molecular cancer therapy with high efficacy and low toxicity [260].

## Conclusion

The three AKT isoforms exert distinct and even opposing roles in tumor growth and metastasis of breast cancer. AKT1 is responsible for proliferation and survival of breast cancer cells, whereas it has an anti-metastatic effect. On the other hand, AKT2 mainly promotes migration, invasion and chemotaxis which are involved in the metastatic process. The effect of AKT3 on breast cancer remains mainly unrevealed, but it seems that AKT3 has an anti-migratory function. The distinct effects of the AKT isoforms are based on different cellular signaling patterns and certain isoform-specific AKT substrates.

The previous classification of AKT as an oncogene could be questioned in regard to even opposing functions of the single AKT isoforms in breast cancer. Even the classification of the various isoforms in oncogene or tumor suppressor seems inappropriate, since the effect of AKT isoforms on tumor growth and metastasis are depending on tumor stage, breast cancer subtype, mutations and probably still unknown influencing factors. As a consequence, it is important to gain further insights into the isoform-specific signaling of AKT in breast cancer. Furthermore, the expression and effect of the single AKT isoforms should be investigated individually for each patient in cell-based assays to determine effective targeted therapies.

## Data Availability

All data presented in this review are included in this publication or in the corresponding original article. The datasets analyzed during the current study are available in the COSMIC database, https://cancer.sanger.ac.uk/cosmic [209]. The signaling pathway in Fig. 1 was generated by using the “Flowchart Designer lite” software.

## Abbreviations

ACG family: Protein kinase A, C and C family
AS160: AKT substrate of 160 kDa
ATP: Adenosine triphosphate
BAD: Bcl-2-associated death promoter
Bcl-XL: B-cell lymphoma-extra large
BDNF: Brain-derived neurotopic factor
Bim: Bcl-2-like protein 11
BRCA1: Breast cancer type 1 susceptibility protein
CDK2/4: Cyclin-dependent kinase 2/4
CK8: Cytokeratin 8
CREB: cAMP response element-binding protein
CXCL-16: C-X-C motif chemokine ligand 16
DCIS: Ductal carcinoma in situ
DDX21: Nucleolar RNA helicase 2
DNA-PK: DNA-dependent protein kinase
DTC/CTC: Disseminated tumor cells / circulating tumor cells
ECM: Extracellular matrix
EGF: Epidermal growth factor
EGFR: Epidermal growth factor receptor
eIF4E: eukaryotic translation initiation factor 4E
EIF4EBP1: Eukaryotic translation initiation factor 4E-binding protein 1
EMT: Epithelial mesenchymal transition
ER(α): Estrogen receptor α
ERK: Extracellular signal–regulated kinases
FAK: Focal adhesion kinase
FoxM1: Forkhead box protein M1
FOXO: Forkhead-box-protein O
GLUT1/4: Glucose transporter type 1/4
GM-CSF: Granulocyte-macrophage colony-stimulating factor
GPCR: G protein-coupled receptor
GSK3: Glycogen synthase kinase 3α/β
Gβγ: G protein, subunits β and γ
HER2/neu: Human epidermal growth factor receptor 2 / ErbB2
IC50: Half maximal inhibitory concentration
IGF-1: Insulin-like growth factor 1
IGF-1R: Insulin-like growth factor 1 receptor
IKK: IKB kinase
LIMK: LIM domain kinase
LPA: Lysophosphatidic acid
MAGI3: Membrane-associated guanylate kinase, WW and PDZ domain containing 3
mDia: protein diaphanous homolog
Mdm2/HDM2: Mouse double minute 2 homolog
MEK/MAPKK: Mitogen-activated protein kinase kinase
MIPγ: Macrophage inflammatory protein-1 γ
miR: micro-RNA
MMP2/9: Matrix metalloproteinase-2/9
MMTV: Mouse mammary tumor virus
MTCP1: Mature T cell proliferation protein 1
mTOR: mammalian target of rapamycin
mTORC1/2: mammalian target of rapamycin complex 1/2
myr-AKT: N-terminally myristoylation signal-attached AKT NFAT1/5: nuclear factor of activated T-cells 1/5
NFKB: Nuclear factor kappa-light-chain-enhancer of activated B cells
NGS: Next generation sequencing
NO: Nitric oxide
NOX4: NADPH oxidase 4
p21: cyclin-dependent kinase inhibitor 1
p27: cyclin-dependent kinase inhibitor 1B
p53: tumor suppressor p53
p70S6K: ribosomal protein S6 kinase beta-1 / p70S6 kinase
Par-1: Protease-activated receptor 1
Par-4/PAWR: Prostate apoptosis response-4 / PRKC, apoptosis, WT1, regulator
PCNA: Proliferating cell nuclear antigen
PDCD4: Programmed cell death protein 4
PDGF: Platelet derived growth factor
PDK1: Phosphoinositide-dependent kinase-1
PGC-1α: Peroxisome proliferator-activated receptor gamma coactivator 1-alpha
PH-domain: Pleckstrin homology domain
PHLPP1/2: PH domain and leucine rich repeat protein phosphatases 1/2
PI(3,4)P2 / PI(4,5)P2: phosphatidylinositol 4,5-bisphosphate / phosphatidylinositol 3,4-bisphosphate
PI(3,4,5)P3: Phosphatidylinositol 3,4,5-trisphosphate
PI3K: Phosphoinositide 3-kinase
PIK3CA: Phosphatidylinositol-4,5-bisphosphate 3-kinase, catalytic subunit alpha
PIKfyve: Phosphoinositide kinase, FYVE type zinc finger containing
PIPP: Inositol polyphosphate 5-phosphatase
PKB: Protein kinase B
PKCγ: Protein kinase C γ
PKCζ: Protein kinase Cζ
PP2A: Protein phosphatase 2
PR: Progesterone receptor
PRAS40: Proline-rich AKT substrate of 40 kDa
PTEN: Phosphatase and tensin homolog
PyMT: Polyomavirus middle T-antigen
Raf: Raf kinase
Rb: Retinoblastoma protein
Rictor: Rapamycin-insensitive companion of mammalian target of rapamycin
ROS: Reactive oxygen species
RTK: Receptor tyrosine kinase
S100A4: S100 calcium-binding protein A4
S473: Serine 473
S6: Ribosomal protein S6
SDF1α: Stromal cell-derived factor 1α
SHIP: SH2-containing inositol phosphatase
SIRT6: Sirtuin 6
Skp2: S-phase kinase-associated protein 2
SNP: Single-nucleotide polymorphism
Sp1: Specificity protein 1
T308: Threonine 308
TCGA: The Cancer Genome Atlas
TCL1/TCL1b: T-cell leukemia/lymphoma protein 1/1b
TGF-β: Transforming growth factor β
TIMP-1: Tissue inhibitor of metalloproteinase-1
TIS21: NGF-inducible protein TIS21
TNBC: Triple negative breast cancer
TNBC: Triple negative breast cancer
TSC2: Tuberous sclerosis complex 2
VEGF: Vascular endothelial growth factor
WDFY2: WD repeat and FYVE domain-containing protein 2
WDR26: WD repeat-containing protein 26
XIAP: X-linked inhibitor of apoptosis protein
Zeb1/2: zinc finger E-box-binding homeobox 1/2

## References

[1] Siegel RL, Miller KD, Jemal A (2019). Cancer statistics, 2019. CA Cancer J Clin.

[2] Lobbezoo DJ, van Kampen RJ, Voogd AC, Dercksen MW, van den Berkmortel F, Smilde TJ (2015). Prognosis of metastatic breast cancer: are there differences between patients with de novo and recurrent metastatic breast cancer?. Br J Cancer.

[3] Parkes A, Clifton K, Al-Awadhi A, Oke O, Warneke CL, Litton JK (2018). Characterization of bone only metastasis patients with respect to tumor subtypes. NPJ Breast Cancer.

[4] Weigelt B, Peterse JL, van 't Veer LJ (2005). Breast cancer metastasis: Markers and models. Nat Rev Cancer.

[5] Hanahan D, Weinberg RA (2000). The hallmarks of cancer. Cell.

[6] Hanahan D, Weinberg RA (2011). Hallmarks of cancer: the next generation. Cell.

[7] Gupta GP, Massague J (2006). Cancer metastasis: Building a framework. Cell.

[8] Altomare DA, Testa JR (2005). Perturbations of the AKT signaling pathway in human cancer. Oncogene.

[9] Vasudevan KM, Garraway LA (2010). AKT signaling in physiology and disease. Curr Top Microbiol Immunol.

[10] Castaneda CA, Cortes-Funes H, Gomez HL, Ciruelos EM (2010). The phosphatidyl inositol 3-kinase/AKT signaling pathway in breast cancer. Cancer Met Rev.

[11] Nicholson KM, Anderson NG (2002). The protein kinase B/Akt signalling pathway in human malignancy. Cell Signal.

[12] Barnett SF, Defeo-Jones D, Fu S, Hancock PJ, Haskell KM, Jones RE (2005). Identification and characterization of pleckstrin-homology-domain-dependent and isoenzyme-specific Akt inhibitors. Biochem J.

[13] DeFeo-Jones D, Barnett SF, Fu S, Hancock PJ, Haskell KM, Leander KR (2005). Tumor cell sensitization to apoptotic stimuli by selective inhibition of specific Akt/PKB family members. Mol Cancer Ther.

[14] Hernandez-Aya LF, Gonzalez-Angulo AM (2011). Targeting the phosphatidylinositol 3-kinase signaling pathway in breast cancer. Oncologist.

[15] Araki K, Miyoshi Y (2018). Mechanism of resistance to endocrine therapy in breast cancer: The important role of PI3K/Akt/mTOR in estrogen receptor-positive, HER2-negative breast cancer. Breast Cancer.

[16] Lopez-Knowles E, O'Toole SA, McNeil CM, Millar EK, Qiu MR, Crea P (2010). PI3K pathway activation in breast cancer is associated with the basal-like phenotype and cancer-specific mortality. Int J Cancer.

[17] Pérez-Tenorio G, Stål O (2002). Activation of AKT/PKB in breast cancer predicts a worse outcome among endocrine treated patients. Br J Cancer.

[18] Chin YR, Toker A (2009). Function of Akt/PKB signaling to cell motility, invasion and the tumor stroma in cancer. Cell Signal.

[19] Bellacosa A, Testa JR, Staal SP, Tsichlis PN (1991). A retroviral oncogene, akt, encoding a serine-threonine kinase containing an SH2-like region. Science.

[20] Coffer PJ, Woodgett JR (1991). Molecular cloning and characterisation of a novel putative protein-serine kinase related to the cAMP-dependent and protein kinase C families. Eur J Biochem.

[21] Jones PF, Jakubowicz T, Pitossi FJ, Maurer F, Hemmings BA (1991). Molecular cloning and identification of a serine/threonine protein kinase of the second-messenger subfamily. Proc Natl Acad Sci U S A.

[22] Staal SP, Hartley JW, Rowe WP (1977). Isolation of transforming murine leukemia viruses from mice with a high incidence of spontaneous lymphoma. Proc Natl Acad Sci U S A.

[23] Hanada M, Feng J, Hemmings BA (2004). Structure, regulation and function of PKB/AKT--a major therapeutic target. Biochim Biophys Acta.

[24] Burgering BM, Coffer PJ (1995). Protein kinase B (c-Akt) in phosphatidylinositol-3-OH kinase signal transduction. Nature.

[25] Okano J, Gaslightwala I, Birnbaum MJ, Rustgi AK, Nakagawa H (2000). Akt/protein kinase B isoforms are differentially regulated by epidermal growth factor stimulation. J Biol Chem.

[26] Olayioye MA, Neve RM, Lane HA, Hynes NE (2000). The ErbB signaling network: receptor heterodimerization in development and cancer. EMBO J.

[27] Alessi DR, Andjelkovic M, Caudwell B, Cron P, Morrice N, Cohen P (1996). Mechanism of activation of protein kinase B by insulin and IGF-1. EMBO J.

[28] Domin J, Waterfield MD (1997). Using structure to define the function of phosphoinositide 3-kinase family members. FEBS Lett.

[29] Hiles ID, Otsu M, Volinia S, Fry MJ, Gout I, Dhand R (1992). Phosphatidylinositol 3-kinase: structure and expression of the 110 kd catalytic subunit. Cell.

[30] Hawkins PT, Jackson TR, Stephens LR (1992). Platelet-derived growth factor stimulates synthesis of PtdIns(3,4,5)P3 by activating a PtdIns(4,5)P2 3-OH kinase. Nature.

[31] Milburn CC, Deak M, Kelly SM, Price NC, Alessi DR, Van Aalten DM (2003). Binding of phosphatidylinositol 3,4,5-trisphosphate to the pleckstrin homology domain of protein kinase B induces a conformational change. Biochem J.

[32] Alessi DR, James SR, Downes CP, Holmes AB, Gaffney PR, Reese CB (1997). Characterization of a 3-phosphoinositide-dependent protein kinase which phosphorylates and activates protein kinase Balpha. Curr Biol.

[33] Bozulic L, Surucu B, Hynx D, Hemmings BA (2008). PKBalpha/Akt1 acts downstream of DNA-PK in the DNA double-strand break response and promotes survival. Mol Cell.

[34] Kawakami Y, Nishimoto H, Kitaura J, Maeda-Yamamoto M, Kato RM, Littman DR (2004). Protein kinase C betaII regulates Akt phosphorylation on Ser-473 in a cell type- and stimulus-specific fashion. J Biol Chem.

[35] Lynch DK, Ellis CA, Edwards PA, Hiles ID (1999). Integrin-linked kinase regulates phosphorylation of serine 473 of protein kinase B by an indirect mechanism. Oncogene.

[36] Toker A, Newton AC (2000). Akt/protein kinase B is regulated by autophosphorylation at the hypothetical PDK-2 site. J Biol Chem.

[37] Stambolic V, Suzuki A, de la Pompa JL, Brothers GM, Mirtsos C, Sasaki T (1998). Negative regulation of PKB/Akt-dependent cell survival by the tumor suppressor PTEN. Cell.

[38] Huber M, Helgason CD, Damen JE, Scheid M, Duronio V, Liu L (1999). The role of SHIP in growth factor induced signalling. Prog Biophys Mol Biol.

[39] Brognard J, Sierecki E, Gao T, Newton AC (2007). PHLPP and a second isoform, PHLPP2, differentially attenuate the amplitude of Akt signaling by regulating distinct Akt isoforms. Mol Cell.

[40] Gao T, Furnari F, Newton AC (2005). PHLPP: a phosphatase that directly dephosphorylates Akt, promotes apoptosis, and suppresses tumor growth. Mol Cell.

[41] Ugi S, Imamura T, Maegawa H, Egawa K, Yoshizaki T, Shi K (2004). Protein phosphatase 2A negatively regulates insulin's metabolic signaling pathway by inhibiting Akt (protein kinase B) activity in 3T3-L1 adipocytes. Mol Cell Biol.

[42] Andjelkovic M, Alessi DR, Meier R, Fernandez A, Lamb NJ, Frech M (1997). Role of translocation in the activation and function of protein kinase B. J Biol Chem.

[43] Ananthanarayanan B, Ni Q, Zhang J (2005). Signal propagation from membrane messengers to nuclear effectors revealed by reporters of phosphoinositide dynamics and Akt activity. Proc Natl Acad Sci U S A.

[44] Obata T, Yaffe MB, Leparc GG, Piro ET, Maegawa H, Kashiwagi A (2000). Peptide and protein library screening defines optimal substrate motifs for AKT/PKB. J Biol Chem.

[45] Manning BD, Cantley LC (2007). AKT/PKB signaling: navigating downstream. Cell.

[46] Kohn AD, Summers SA, Birnbaum MJ, Roth RA (1996). Expression of a constitutively active Akt Ser/Thr kinase in 3T3-L1 adipocytes stimulates glucose uptake and glucose transporter 4 translocation. J Biol Chem.

[47] Sano H, Kane S, Sano E, Miinea CP, Asara JM, Lane WS (2003). Insulin-stimulated phosphorylation of a Rab GTPase-activating protein regulates GLUT4 translocation. J Biol Chem.

[48] Berwick DC, Dell GC, Welsh GI, Heesom KJ, Hers I, Fletcher LM (2004). Protein kinase B phosphorylation of PIKfyve regulates the trafficking of GLUT4 vesicles. J Cell Sci.

[49] Diehl JA, Cheng M, Roussel MF, Sherr CJ (1998). Glycogen synthase kinase-3beta regulates cyclin D1 proteolysis and subcellular localization. Genes Dev.

[50] Cross DA, Alessi DR, Cohen P, Andjelkovich M, Hemmings BA (1995). Inhibition of glycogen synthase kinase-3 by insulin mediated by protein kinase B. Nature.

[51] Liang J, Zubovitz J, Petrocelli T, Kotchetkov R, Connor MK, Han K (2002). PKB/Akt phosphorylates p27, impairs nuclear import of p27 and opposes p27-mediated G1 arrest. Nat Med.

[52] Zhou BP, Liao Y, Xia W, Spohn B, Lee MH, Hung MC (2001). Cytoplasmic localization of p21Cip1/WAF1 by Akt-induced phosphorylation in HER-2/neu-overexpressing cells. Nat Cell Biol.

[53] Zhou BP, Liao Y, Xia W, Zou Y, Spohn B, Hung MC (2001). HER-2/neu induces p53 ubiquitination via Akt-mediated MDM2 phosphorylation. Nat Cell Biol.

[54] Datta SR, Dudek H, Tao X, Masters S, Fu H, Gotoh Y (1997). Akt phosphorylation of BAD couples survival signals to the cell-intrinsic death machinery. Cell.

[55] Cardone MH, Roy N, Stennicke HR, Salvesen GS, Franke TF, Stanbridge E (1998). Regulation of cell death protease caspase-9 by phosphorylation. Science.

[56] Brunet A, Bonni A, Zigmond MJ, Lin MZ, Juo P, Hu LS (1999). Akt promotes cell survival by phosphorylating and inhibiting a Forkhead transcription factor. Cell.

[57] Ozes ON, Mayo LD, Gustin JA, Pfeffer SR, Pfeffer LM, Donner DB (1999). NF-kappaB activation by tumour necrosis factor requires the Akt serine-threonine kinase. Nature.

[58] Enomoto A, Murakami H, Asai N, Morone N, Watanabe T, Kawai K (2005). Akt/PKB regulates actin organization and cell motility via Girdin/APE. Dev Cell.

[59] Park BK, Zeng X, Glazer RI (2001). Akt1 induces extracellular matrix invasion and matrix metalloproteinase-2 activity in mouse mammary epithelial cells. Cancer Res.

[60] Kim D, Kim S, Koh H, Yoon SO, Chung AS, Cho KS (2001). Akt/PKB promotes cancer cell invasion via increased motility and metalloproteinase production. FASEB J.

[61] Tee AR, Fingar DC, Manning BD, Kwiatkowski DJ, Cantley LC, Blenis J (2002). Tuberous sclerosis complex-1 and -2 gene products function together to inhibit mammalian target of rapamycin (mTOR)-mediated downstream signaling. Proc Natl Acad Sci U S A.

[62] Inoki K, Li Y, Zhu T, Wu J, Guan KL (2002). TSC2 is phosphorylated and inhibited by Akt and suppresses mTOR signalling. Nat Cell Biol.

[63] Vander Haar E, Lee SI, Bandhakavi S, Griffin TJ, Kim DH (2007). Insulin signalling to mTOR mediated by the Akt/PKB substrate PRAS40. Nat Cell Biol.

[64] Yang SX, Polley E, Lipkowitz S (2016). New insights on PI3K/AKT pathway alterations and clinical outcomes in breast cancer. Cancer Treat Rev.

[65] Riggio M, Polo ML, Blaustein M, Colman-Lerner A, Luthy I, Lanari C (2012). PI3K/AKT pathway regulates phosphorylation of steroid receptors, hormone independence and tumor differentiation in breast cancer. Carcinogenesis.

[66] Lee YR, Park J, Yu HN, Kim JS, Youn HJ, Jung SH (2005). Up-regulation of PI3K/Akt signaling by 17beta-estradiol through activation of estrogen receptor-alpha, but not estrogen receptor-beta, and stimulates cell growth in breast cancer cells. Biochem Biophys Res Commun.

[67] Qiao M, Sheng S, Pardee AB (2008). Metastasis and AKT activation. Cell Cycle.

[68] Elloul S, Kedrin D, Knoblauch NW, Beck AH, Toker A (2014). The adherens junction protein afadin is an AKT substrate that regulates breast cancer cell migration. Mol Cancer Res.

[69] Perez-Tenorio G, Alkhori L, Olsson B, Waltersson MA, Nordenskjold B, Rutqvist LE (2007). PIK3CA mutations and PTEN loss correlate with similar prognostic factors and are not mutually exclusive in breast cancer. Clin Cancer Res.

[70] Kalinsky K, Jacks LM, Heguy A, Patil S, Drobnjak M, Bhanot UK (2009). PIK3CA mutation associates with improved outcome in breast cancer. Clin Cancer Res.

[71] Stemke-Hale K, Gonzalez-Angulo AM, Lluch A, Neve RM, Kuo W-L, Davies M (2008). An integrative genomic and proteomic analysis of PIK3CA, PTEN, and AKT mutations in breast cancer. Cancer Res.

[72] Bose S, Crane A, Hibshoosh H, Mansukhani M, Sandweis L, Parsons R (2002). Reduced expression of PTEN correlates with breast cancer progression. Hum Pathol.

[73] Slamon DJ, Godolphin W, Jones LA, Holt JA, Wong SG, Keith DE (1989). Studies of the HER-2/neu proto-oncogene in human breast and ovarian cancer. Science.

[74] Carpten JD, Faber AL, Horn C, Donoho GP, Briggs SL, Robbins CM (2007). A transforming mutation in the pleckstrin homology domain of AKT1 in cancer. Nature.

[75] Bellacosa A (1995). Feo Dd, Godwin AK, Bell DW, Cheng JQ, Altomare DA, et al. Molecular alterations of the AKT2 oncogene in ovarian and breast carcinomas. Int J Cancer.

[76] Onitilo AA, Engel JM, Greenlee RT, Mukesh BN (2009). Breast cancer subtypes based on ER/PR and Her2 expression: comparison of clinicopathologic features and survival. Clin Med Res.

[77] Renner O, Blanco-Aparicio C, Grassow M, Cañamero M, Leal JFM, Carnero A (2008). Activation of phosphatidylinositol 3-kinase by membrane localization of p110alpha predisposes mammary glands to neoplastic transformation. Cancer Res.

[78] Schmitz KJ, Otterbach F, Callies R, Levkau B, Holscher M, Hoffmann O (2004). Prognostic relevance of activated Akt kinase in node-negative breast cancer: a clinicopathological study of 99 cases. Mod Pathol.

[79] Li X, Yang Q, Yu H, Wu L, Zhao Y, Zhang C (2014). LIF promotes tumorigenesis and metastasis of breast cancer through the AKT-mTOR pathway. Oncotarget.

[80] Clark AS, West K, Streicher S, Dennis PA (2002). Constitutive and inducible Akt activity promotes resistance to chemotherapy, trastuzumab, or tamoxifen in breast cancer cells. Mol Cancer Ther.

[81] Kirkegaard T, Witton CJ, McGlynn LM, Tovey SM, Dunne B, Lyon A (2005). AKT activation predicts outcome in breast cancer patients treated with tamoxifen. J Pathol..

[82] Stål O, Pérez-Tenorio G, Akerberg L, Olsson B, Nordenskjöld B, Skoog L (2003). Akt kinases in breast cancer and the results of adjuvant therapy. Breast Cancer Res.

[83] Andre F, Nahta R, Conforti R, Boulet T, Aziz M, Yuan LX (2008). Expression patterns and predictive value of phosphorylated AKT in early-stage breast cancer. Ann Oncol.

[84] Yang SX, Costantino JP, Kim C, Mamounas EP, Nguyen D, Jeong JH (2010). Akt phosphorylation at Ser473 predicts benefit of paclitaxel chemotherapy in node-positive breast cancer. J Clin Oncol.

[85] Gupta AK, Cerniglia GJ, Mick R, Ahmed MS, Bakanauskas VJ, Muschel RJ (2003). Radiation sensitization of human cancer cells in vivo by inhibiting the activity of PI3K using LY294002. Int J Radiat Oncol Biol Phys.

[86] Yardley DA, Noguchi S, Pritchard KI, Burris HA, Baselga J, Gnant M (2013). Everolimus plus exemestane in postmenopausal patients with HR(+) breast cancer: BOLERO-2 final progression-free survival analysis. Adv Ther.

[87] Hirai H, Sootome H, Nakatsuru Y, Miyama K, Taguchi S, Tsujioka K (2010). MK-2206, an allosteric Akt inhibitor, enhances antitumor efficacy by standard chemotherapeutic agents or molecular targeted drugs in vitro and in vivo. Mol Cancer Ther.

[88] Yap TA, Yan L, Patnaik A, Fearen I, Olmos D, Papadopoulos K (2011). First-in-man clinical trial of the oral pan-AKT inhibitor MK-2206 in patients with advanced solid tumors. J Clin Oncol.

[89] Sangai T, Akcakanat A, Chen H, Tarco E, Wu Y, Do KA (2012). Biomarkers of response to Akt inhibitor MK-2206 in breast cancer. Clin Cancer Res.

[90] Wisinski KB, Tevaarwerk AJ, Burkard ME, Rampurwala M, Eickhoff J, Bell MC (2016). Phase I Study of an AKT Inhibitor (MK-2206) Combined with Lapatinib in Adult Solid Tumors Followed by Dose Expansion in Advanced HER2+ Breast Cancer. Clin Cancer Res.

[91] Gills JJ, Dennis PA (2009). Perifosine: update on a novel Akt inhibitor. Curr Oncol Rep.

[92] Kumar CC, Madison V (2005). AKT crystal structure and AKT-specific inhibitors. Oncogene.

[93] Jones PF, Jakubowicz T, Hemmings BA (1991). Molecular cloning of a second form of rac protein kinase. Cell Regul.

[94] Konishi H, Kuroda S, Tanaka M, Matsuzaki H, Ono Y, Kameyama K (1995). Molecular cloning and characterization of a new member of the RAC protein kinase family: association of the pleckstrin homology domain of three types of RAC protein kinase with protein kinase C subspecies and beta gamma subunits of G proteins. Biochem Biophys Res Commun.

[95] Brodbeck D, Cron P, Hemmings BA (1999). A human protein kinase Bgamma with regulatory phosphorylation sites in the activation loop and in the C-terminal hydrophobic domain. J Biol Chem.

[96] Suyama K, Yao J, Liang H, Benard O, Loudig OD, Amgalan D (2018). An Akt3 Splice Variant Lacking the Serine 472 Phosphorylation Site Promotes Apoptosis and Suppresses Mammary Tumorigenesis. Cancer Res.

[97] Staal SP, Huebner K, Croce CM, Parsa NZ, Testa JR (1988). The AKT1 proto-oncogene maps to human chromosome 14, band q32. Genomics.

[98] Cheng JQ, Godwin AK, Bellacosa A, Taguchi T, Franke TF, Hamilton TC (1992). AKT2, a putative oncogene encoding a member of a subfamily of protein-serine/threonine kinases, is amplified in human ovarian carcinomas. Proc Natl Acad Sci U S A.

[99] Nakatani K, Thompson DA, Barthel A, Sakaue H, Liu W, Weigel RJ (1999). Up-regulation of Akt3 in Estrogen Receptor-deficient Breast Cancers and Androgen-independent Prostate Cancer Lines. J Biol Chem.

[100] O'Hurley G, Daly E, O'Grady A, Cummins R, Quinn C, Flanagan L (2014). Investigation of molecular alterations of AKT-3 in triple-negative breast cancer. Histopathology.

[101] Pérez-Tenorio G, Karlsson E, Stål O (2014). Clinical value of isoform-specific detection and targeting of AKT1, AKT2 and AKT3 in breast cancer. Breast Cancer Man.

[102] Chen WS, Xu PZ, Gottlob K, Chen ML, Sokol K, Shiyanova T (2001). Growth retardation and increased apoptosis in mice with homozygous disruption of the Akt1 gene. Genes Dev.

[103] Garofalo RS, Orena SJ, Rafidi K, Torchia AJ, Stock JL, Hildebrandt AL (2003). Severe diabetes, age-dependent loss of adipose tissue, and mild growth deficiency in mice lacking Akt2/PKB beta. J Clin Invest.

[104] Cho H, Mu J, Kim JK, Thorvaldsen JL, Chu Q, Crenshaw EB (2001). Insulin resistance and a diabetes mellitus-like syndrome in mice lacking the protein kinase Akt2 (PKB beta). Science.

[105] Tschopp O, Yang Z-Z, Brodbeck D, Dummler BA, Hemmings-Mieszczak M, Watanabe T (2005). Essential role of protein kinase B gamma (PKB gamma/Akt3) in postnatal brain development but not in glucose homeostasis. Development.

[106] Easton RM, Cho H, Roovers K, Shineman DW, Mizrahi M, Forman MS (2005). Role for Akt3/protein kinase Bgamma in attainment of normal brain size. Mol Cell Biol.

[107] Peng XD, Xu PZ, Chen ML, Hahn-Windgassen A, Skeen J, Jacobs J (2003). Dwarfism, impaired skin development, skeletal muscle atrophy, delayed bone development, and impeded adipogenesis in mice lacking Akt1 and Akt2. Genes Dev.

[108] Yang ZZ, Tschopp O, Di-Poi N, Bruder E, Baudry A, Dummler B (2005). Dosage-dependent effects of Akt1/protein kinase Balpha (PKBalpha) and Akt3/PKBgamma on thymus, skin, and cardiovascular and nervous system development in mice. Mol Cell Biol.

[109] Dummler B, Tschopp O, Hynx D, Yang ZZ, Dirnhofer S, Hemmings BA (2006). Life with a single isoform of Akt: mice lacking Akt2 and Akt3 are viable but display impaired glucose homeostasis and growth deficiencies. Mol Cell Biol.

[110] Cheung M, Testa JR (2013). Diverse mechanisms of AKT pathway activation in human malignancy. Curr Cancer Drug Targets.

[111] Loi S, Haibe-Kains B, Majjaj S, Lallemand F, Durbecq V, Larsimont D (2010). PIK3CA mutations associated with gene signature of low mTORC1 signaling and better outcomes in estrogen receptor-positive breast cancer. Proc Natl Acad Sci U S A.

[112] Yoeli-Lerner M, Yiu GK, Rabinovitz I, Erhardt P, Jauliac S, Toker A (2005). Akt blocks breast cancer cell motility and invasion through the transcription factor NFAT. Mol Cell.

[113] Li W, Hou J-Z, Niu J, Xi Z-Q, Ma C, Sun H (2018). Akt1 inhibition promotes breast cancer metastasis through EGFR-mediated β-catenin nuclear accumulation. Cell Commun Signal.

[114] Watson CJ (2006). Key stages in mammary gland development - Involution: apoptosis and tissue remodelling that convert the mammary gland from milk factory to a quiescent organ. Breast Cancer Res.

[115] Walker NI, Bennett RE, Kerr JF (1989). Cell death by apoptosis during involution of the lactating breast in mice and rats. Am J Anat..

[116] Schwertfeger KL, Richert MM, Anderson SM (2001). Mammary gland involution is delayed by activated Akt in transgenic mice. Mol Endocrinol.

[117] Hutchinson J, Jin J, Cardiff RD, Woodgett JR, Muller WJ (2001). Activation of Akt (protein kinase B) in mammary epithelium provides a critical cell survival signal required for tumor progression. Mol Cell Biol.

[118] Ackler S, Ahmad S, Tobias C, Johnson MD, Glazer RI (2002). Delayed mammary gland involution in MMTV-AKT1 transgenic mice. Oncogene.

[119] Blanco-Aparicio C, Pérez-Gallego L, Pequeño B, Leal JFM, Renner O, Carnero A (2007). Mice expressing myrAKT1 in the mammary gland develop carcinogen-induced ER-positive mammary tumors that mimic human breast cancer. Carcinogenesis.

[120] Boxer RB, Stairs DB, Dugan KD, Notarfrancesco KL, Portocarrero CP, Keister BA (2006). Isoform-specific requirement for Akt1 in the developmental regulation of cellular metabolism during lactation. Cell Metab.

[121] Baek HJ, Kim SE, Kim JK, Shin DH, Kim TH, Kim KG (2018). Inhibition of AKT suppresses the initiation and progression of BRCA1-associated mammary tumors. Int J Biol Sci.

[122] Maroulakou IG, Oemler W, Naber SP, Klebba I, Kuperwasser C, Tsichlis PN (2008). Distinct roles of the three Akt isoforms in lactogenic differentiation and involution. J Cell Physiol.

[123] Dillon RL, Marcotte R, Hennessy BT, Woodgett JR, Mills GB, Muller WJ (2009). Akt1 and akt2 play distinct roles in the initiation and metastatic phases of mammary tumor progression. Cancer Res.

[124] Maroulakou IG, Oemler W, Naber SP, Tsichlis PN (2007). Akt1 ablation inhibits, whereas Akt2 ablation accelerates, the development of mammary adenocarcinomas in mouse mammary tumor virus (MMTV)-ErbB2/neu and MMTV-polyoma middle T transgenic mice. Cancer Res.

[125] Hutchinson JN, Jin J, Cardiff RD, Woodgett JR, Muller WJ (2004). Activation of Akt-1 (PKB-alpha) can accelerate ErbB-2-mediated mammary tumorigenesis but suppresses tumor invasion. Cancer Res.

[126] Yang W (2011). Ju J-h, Lee K-m, Shin I. Akt isoform-specific inhibition of MDA-MB-231 cell proliferation. Cell Signal.

[127] Riggio M, Perrone MC, Polo ML, Rodriguez MJ, May M, Abba M (2017). AKT1 and AKT2 isoforms play distinct roles during breast cancer progression through the regulation of specific downstream proteins. Sci Rep.

[128] Santi SA, Lee H (2011). Ablation of Akt2 induces autophagy through cell cycle arrest, the downregulation of p70S6K, and the deregulation of mitochondria in MDA-MB231 cells. PLoS ONE.

[129] Liu H, Radisky DC, Nelson CM, Zhang H, Fata JE, Roth RA (2006). Mechanism of Akt1 inhibition of breast cancer cell invasion reveals a protumorigenic role for TSC2. Proc Natl Acad Sci U S A.

[130] Grabinski N, Möllmann K, Milde-Langosch K, Müller V, Schumacher U, Brandt B (2014). AKT3 regulates ErbB2, ErbB3 and estrogen receptor α expression and contributes to endocrine therapy resistance of ErbB2(+) breast tumor cells from Balb-neuT mice. Cell Signal.

[131] Toulany M, Maier J, Iida M, Rebholz S, Holler M, Grottke A (2017). Akt1 and Akt3 but not Akt2 through interaction with DNA-PKcs stimulate proliferation and post-irradiation cell survival of K-RAS-mutated cancer cells. Cell Death Discov.

[132] Stottrup C, Tsang T, Chin YR (2016). Upregulation of AKT3 Confers Resistance to the AKT Inhibitor MK2206 in Breast Cancer. Mol Cancer Ther..

[133] Chin YR, Yuan X, Balk SP, Toker A (2014). PTEN-deficient tumors depend on AKT2 for maintenance and survival. Cancer Discov.

[134] Héron-Milhavet L, Franckhauser C, Rana V, Berthenet C, Fisher D, Hemmings BA (2006). Only Akt1 is required for proliferation, while Akt2 promotes cell cycle exit through p21 binding. Mol Cell Biol.

[135] Gao D, Inuzuka H, Tseng A, Chin RY, Toker A, Wei W (2009). Phosphorylation by Akt1 promotes cytoplasmic localization of Skp2 and impairs APCCdh1-mediated Skp2 destruction. Nat Cell Biol.

[136] Ju X, Katiyar S, Wang C, Liu M, Jiao X, Li S (2007). Akt1 governs breast cancer progression in vivo. Proc Natl Acad Sci U S A.

[137] Wang J, Wan W, Sun R, Liu Y, Sun X, Ma D (2008). Reduction of Akt2 expression inhibits chemotaxis signal transduction in human breast cancer cells. Cell Signal.

[138] Zhang J, Li G, Chen Y, Fang L, Guan C, Bai F (2017). Metformin Inhibits Tumorigenesis and Tumor Growth of Breast Cancer Cells by Upregulating miR-200c but Downregulating AKT2 Expression. J Cancer.

[139] Polytarchou C, Iliopoulos D, Hatziapostolou M, Kottakis F, Maroulakou I, Struhl K (2011). Akt2 regulates all Akt isoforms and promotes resistance to hypoxia through induction of miR-21 upon oxygen deprivation. Cancer Res.

[140] Ye Y, Tang X, Sun Z, Chen S (2016). Upregulated WDR26 serves as a scaffold to coordinate PI3K/ AKT pathway-driven breast cancer cell growth, migration, and invasion. Oncotarget.

[141] Yu Z, Xu Z, Disante G, Wright J, Wang M, Li Y (2014). miR-17/20 sensitization of breast cancer cells to chemotherapy-induced apoptosis requires Akt1. Oncotarget.

[142] Thirumurthi U, Shen J, Xia W, LaBaff AM, Wei Y, Li CW (2014). MDM2-mediated degradation of SIRT6 phosphorylated by AKT1 promotes tumorigenesis and trastuzumab resistance in breast cancer. Sci Signal.

[143] Plo I, Laulier C, Gauthier L, Lebrun F, Calvo F, Lopez BS (2008). AKT1 inhibits homologous recombination by inducing cytoplasmic retention of BRCA1 and RAD51. Cancer Res.

[144] Ooms LM, Binge LC, Davies EM, Rahman P, Conway JRW, Gurung R (2015). The Inositol Polyphosphate 5-Phosphatase PIPP Regulates AKT1-Dependent Breast Cancer Growth and Metastasis. Cancer Cell.

[145] Zhang G, Liu Z, Xu H, Yang Q (2016). miR-409-3p suppresses breast cancer cell growth and invasion by targeting Akt1. Biochem Biophys Res Commun.

[146] Yang E, Boire A, Agarwal A, Nguyen N, O'Callaghan K, Tu P (2009). Blockade of PAR1 signaling with cell-penetrating pepducins inhibits Akt survival pathways in breast cancer cells and suppresses tumor survival and metastasis. Cancer Res.

[147] Watson KL, Moorehead RA (2013). Loss of Akt1 or Akt2 delays mammary tumor onset and suppresses tumor growth rate in MTB-IGFIR transgenic mice. BMC Cancer.

[148] Irie HY, Pearline RV, Grueneberg D, Hsia M, Ravichandran P, Kothari N (2005). Distinct roles of Akt1 and Akt2 in regulating cell migration and epithelial-mesenchymal transition. J Cell Biol.

[149] Gargini R, Cerliani JP, Escoll M, Anton IM, Wandosell F (2015). Cancer stem cell-like phenotype and survival are coordinately regulated by Akt/FoxO/Bim pathway. Stem Cells.

[150] Grottke A, Ewald F, Lange T, Nörz D, Herzberger C, Bach J (2016). Downregulation of AKT3 Increases Migration and Metastasis in Triple Negative Breast Cancer Cells by Upregulating S100A4. PLoS ONE.

[151] Chin YR, Yoshida T, Marusyk A, Beck AH, Polyak K, Toker A (2014). Targeting Akt3 signaling in triple-negative breast cancer. Cancer Res.

[152] Choi JA, Jung YS, Kim JY, Kim HM, Lim IK (2016). Inhibition of breast cancer invasion by TIS21/BTG2/Pc3-Akt1-Sp1-Nox4 pathway targeting actin nucleators, mDia genes. Oncogene.

[153] Faridi J, Wang L, Endemann G, Roth RA (2003). Expression of constitutively active Akt-3 in MCF-7 breast cancer cells reverses the estrogen and tamoxifen responsivity of these cells in vivo. Clin Cancer Res.

[154] Chung S, Yao J, Suyama K, Bajaj S, Qian X, Loudig OD (2013). N-cadherin regulates mammary tumor cell migration through Akt3 suppression. Oncogene.

[155] Hu X, Wang J, He W, Zhao P, Ye C (2018). MicroRNA-433 targets AKT3 and inhibits cell proliferation and viability in breast cancer. Oncol Lett..

[156] Li Y, Cai B, Shen L, Dong Y, Lu Q, Sun S (2017). MiRNA-29b suppresses tumor growth through simultaneously inhibiting angiogenesis and tumorigenesis by targeting Akt3. Cancer Lett.

[157] Lehman HL, van Laere SJ, van Golen CM, Vermeulen PB, Dirix LY, van Golen KL (2012). Regulation of inflammatory breast cancer cell invasion through Akt1/PKBα phosphorylation of RhoC GTPase. Mol Cancer Res.

[158] Chin YR, Toker A (2010). Akt2 regulates expression of the actin-bundling protein palladin. FEBS Lett.

[159] Chin YR, Toker A (2010). The actin-bundling protein palladin is an Akt1-specific substrate that regulates breast cancer cell migration. Mol Cell.

[160] Chin YR, Toker A (2014). Akt isoform-specific signaling in breast cancer: uncovering an anti-migratory role for palladin. Cell Adh Migr.

[161] Yoeli-Lerner M, Chin YR, Hansen CK, Toker A (2009). Akt/protein kinase b and glycogen synthase kinase-3beta signaling pathway regulates cell migration through the NFAT1 transcription factor. Mol Cancer Res.

[162] Steelman LS, Navolanic P, Chappell WH, Abrams SL, Wong EW, Martelli AM (2011). Involvement of Akt and mTOR in chemotherapeutic- and hormonal-based drug resistance and response to radiation in breast cancer cells. Cell Cycle.

[163] Iliopoulos D, Polytarchou C, Hatziapostolou M, Kottakis F, Maroulakou IG, Struhl K (2009). MicroRNAs differentially regulated by Akt isoforms control EMT and stem cell renewal in cancer cells. Sci Signal.

[164] Cheng GZ, Chan J, Wang Q, Zhang W, Sun CD, Wang L-H (2007). Twist transcriptionally up-regulates AKT2 in breast cancer cells leading to increased migration, invasion, and resistance to paclitaxel. Cancer Res.

[165] Leal-Orta Elizabeth, Ramirez-Ricardo Javier, Cortes-Reynosa Pedro, Galindo-Hernandez Octavio, Salazar Eduardo Perez (2018). Role of PI3K/Akt on migration and invasion of MCF10A cells treated with extracellular vesicles from MDA-MB-231 cells stimulated with linoleic acid. Journal of Cell Communication and Signaling.

[166] Marcial-Medina Cleofas, Ordoñez-Moreno Alejandra, Gonzalez-Reyes Christian, Cortes-Reynosa Pedro, Perez Salazar Eduardo (2019). Oleic acid induces migration through a FFAR1/4, EGFR and AKT-dependent pathway in breast cancer cells. Endocrine Connections.

[167] Arboleda MJ, Lyons JF, Kabbinavar FF, Bray MR, Snow BE, Ayala R (2003). Overexpression of AKT2/protein kinase Bbeta leads to up-regulation of beta1 integrins, increased invasion, and metastasis of human breast and ovarian cancer cells. Cancer Res.

[168] Cheng GZ, Zhang W, Wang L-H (2008). Regulation of cancer cell survival, migration, and invasion by Twist: AKT2 comes to interplay. Cancer Res.

[169] Wang J, Zhao W, Guo H, Fang Y, Stockman SE, Bai S (2018). AKT isoform-specific expression and activation across cancer lineages. BMC Cancer.

[170] Li C-W, Xia W, Lim S-O, Hsu JL, Huo L, Wu Y (2016). AKT1 Inhibits Epithelial-to-Mesenchymal Transition in Breast Cancer through Phosphorylation-Dependent Twist1 Degradation. Cancer Res.

[171] Hohensee I, Chuang H-N, Grottke A, Werner S, Schulte A, Horn S (2017). PTEN mediates the cross talk between breast and glial cells in brain metastases leading to rapid disease progression. Oncotarget.

[172] Joglekar M, Elbezanti WO, Weitzman MD, Lehman HL, van Golen KL (2015). Caveolin-1 mediates inflammatory breast cancer cell invasion via the Akt1 pathway and RhoC GTPase. J Cell Biochem.

[173] Ackah E, Yu J, Zoellner S, Iwakiri Y, Skurk C, Shibata R (2005). Akt1/protein kinase Balpha is critical for ischemic and VEGF-mediated angiogenesis. J Clin Invest.

[174] Lin H-JL, Zuo T, Lin C-H, Kuo CT, Liyanarachchi S, Sun S (2008). Breast cancer-associated fibroblasts confer AKT1-mediated epigenetic silencing of Cystatin M in epithelial cells. Cancer Res.

[175] Pereira L, Horta S, Mateus R, Videira MA (2015). Implications of Akt2/Twist crosstalk on breast cancer metastatic outcome. Drug Discov Today.

[176] Rivas Sergio, Gómez-Oro Carla, Antón Inés, Wandosell Francisco (2018). Role of Akt Isoforms Controlling Cancer Stem Cell Survival, Phenotype and Self-Renewal. Biomedicines.

[177] Park S, Song J, Joe CO, Shin I (2008). Akt stabilizes estrogen receptor alpha with the concomitant reduction in its transcriptional activity. Cell Signal.

[178] Ahmad S, Singh N, Glazer RI (1999). Role of AKT1 in 17β-estradiol- and insulin-like growth factor I (IGF-I)-dependent proliferation and prevention of apoptosis in MCF-7 breast carcinoma cells. Biochem Pharmacol.

[179] Sun M, Paciga JE, Feldman RI, Yuan Z, Coppola D, Lu YY (2001). Phosphatidylinositol-3-OH Kinase (PI3K)/AKT2, activated in breast cancer, regulates and is induced by estrogen receptor alpha (ERalpha) via interaction between ERalpha and PI3K. Cancer Res.

[180] Morelli C, Lanzino M, Garofalo C, Maris P, Brunelli E, Casaburi I (2010). Akt2 inhibition enables the forkhead transcription factor FoxO3a to have a repressive role in estrogen receptor alpha transcriptional activity in breast cancer cells. Mol Cell Biol.

[181] Nitulescu GM, Margina D, Juzenas P, Peng Q, Olaru OT, Saloustros E (2016). Akt inhibitors in cancer treatment: The long journey from drug discovery to clinical use (Review). Int J Oncol.

[182] She Q-B, Chandarlapaty S, Ye Q, Lobo J, Haskell KM, Leander KR (2008). Breast tumor cells with PI3K mutation or HER2 amplification are selectively addicted to Akt signaling. PLoS ONE.

[183] Vasudevan KM, Barbie DA, Davies MA, Rabinovsky R, McNear CJ, Kim JJ (2009). AKT-independent signaling downstream of oncogenic PIK3CA mutations in human cancer. Cancer Cell.

[184] Heiser LM, Sadanandam A, Kuo W-L, Benz SC, Goldstein TC, Ng S (2012). Subtype and pathway specific responses to anticancer compounds in breast cancer. Proc Natl Acad Sci U S A..

[185] Hyman DM, Smyth LM, Donoghue MTA, Westin SN, Bedard PL, Dean EJ (2017). AKT Inhibition in Solid Tumors With AKT1 Mutations. J Clin Oncol.

[186] Bilodeau MT, Balitza AE, Hoffman JM, Manley PJ, Barnett SF, Defeo-Jones D (2008). Allosteric inhibitors of Akt1 and Akt2: a naphthyridinone with efficacy in an A2780 tumor xenograft model. Bioorg Med Chem Lett.

[187] Li Y, Liang J, Siu T, Hu E, Rossi MA, Barnett SF (2009). Allosteric inhibitors of Akt1 and Akt2: discovery of [1,2,4]triazolo[3,4-f][1,6] naphthyridines with potent and balanced activity. Bioorg Med Chem Lett.

[188] Guo D-D, Hong S-H, Jiang H-L, Kim J-H, Minai-Tehrani A, Kim J-E (2012). Synergistic effects of Akt1 shRNA and paclitaxel-incorporated conjugated linoleic acid-coupled poloxamer thermosensitive hydrogel on breast cancer. Biomaterials.

[189] Salhia B, Van Cott C, Tegeler T, Polpitiya A, Duquette RA, Gale M (2012). Differential effects of AKT1(p.E17K) expression on human mammary luminal epithelial and myoepithelial cells. Hum Mutat.

[190] Lauring J, Cosgrove DP, Fontana S, Gustin JP, Konishi H, Abukhdeir AM (2010). Knock in of the AKT1 E17K mutation in human breast epithelial cells does not recapitulate oncogenic PIK3CA mutations. Oncogene.

[191] Beaver JA, Gustin JP, Yi KH, Rajpurohit A, Thomas M, Gilbert SF (2013). PIK3CA and AKT1 mutations have distinct effects on sensitivity to targeted pathway inhibitors in an isogenic luminal breast cancer model system. Clin Cancer Res.

[192] Gonzalez E, McGraw TE (2009). Insulin-modulated Akt subcellular localization determines Akt isoform-specific signaling. Proc Natl Acad Sci U S A.

[193] Iacovides DC, Johnson AB, Wang N, Boddapati S, Korkola J, Gray JW (2013). Identification and quantification of AKT isoforms and phosphoforms in breast cancer using a novel nanofluidic immunoassay. Mol Cell Proteomics.

[194] Santi SA, Lee H (2010). The Akt isoforms are present at distinct subcellular locations. Am J Physiol Cell Physiol.

[195] Spears M, Cunningham CA, Taylor KJ, Mallon EA, Thomas JS, Kerr GR (2012). Proximity ligation assays for isoform-specific Akt activation in breast cancer identify activated Akt1 as a driver of progression. J Pathol.

[196] Plant HC, Kashyap AS, Manton KJ, Hollier BG, Hurst CP, Stein SR (2014). Differential subcellular and extracellular localisations of proteins required for insulin-like growth factor- and extracellular matrix-induced signalling events in breast cancer progression. BMC Cancer.

[197] Sun M, Wang G, Paciga JE, Feldman RI, Yuan Z-Q, Ma X-L (2001). AKT1/PKBα Kinase Is Frequently Elevated in Human Cancers and Its Constitutive Activation Is Required for Oncogenic Transformation in NIH3T3 Cells. Am J Pathol.

[198] van Agthoven T, Sieuwerts AM, Meijer-van Gelder ME, Look MP, Smid M, Veldscholte J (2009). Relevance of breast cancer antiestrogen resistance genes in human breast cancer progression and tamoxifen resistance. J Clin Oncol.

[199] Zinda MJ, Johnson MA, Paul JD, Horn C, Konicek BW, Lu ZH (2001). AKT-1, −2, and −3 are expressed in both normal and tumor tissues of the lung, breast, prostate, and colon. Clin Cancer Res.

[200] Kirkegaard T, Witton CJ, Edwards J, Nielsen KV, Jensen LB, Campbell FM (2010). Molecular alterations in AKT1, AKT2 and AKT3 detected in breast and prostatic cancer by FISH. Histopathology.

[201] Grell P, Fabian P, Khoylou M, Radova L, Slaby O, Hrstka R (2012). Akt expression and compartmentalization in prediction of clinical outcome in HER2-positive metastatic breast cancer patients treated with trastuzumab. Int J Oncol.

[202] Carmona FJ, Montemurro F, Kannan S, Rossi V, Verma C, Baselga J (2016). AKT signaling in ERBB2-amplified breast cancer. Pharmacol Ther.

[203] Bacus SS, Altomare DA, Lyass L, Chin DM, Farrell MP, Gurova K (2002). AKT2 is frequently upregulated in HER-2/neu-positive breast cancers and may contribute to tumor aggressiveness by enhancing cell survival. Oncogene.

[204] Florena AM, Tripodo C, Guarnotta C, Ingrao S, Porcasi R, Martorana A (2007). Associations between Notch-2, Akt-1 and HER2/neu expression in invasive human breast cancer: A tissue microarray immunophenotypic analysis on 98 patients. Pathobiology.

[205] Cancer Genome Atlas N (2012). Comprehensive molecular portraits of human breast tumours. Nature.

[206] Grabinski N, Bartkowiak K, Grupp K, Brandt B, Pantel K, Jücker M (2011). Distinct functional roles of Akt isoforms for proliferation, survival, migration and EGF-mediated signalling in lung cancer derived disseminated tumor cells. Cell Signal.

[207] Aktas B, Tewes M, Fehm T, Hauch S, Kimmig R, Kasimir-Bauer S (2009). Stem cell and epithelial-mesenchymal transition markers are frequently overexpressed in circulating tumor cells of metastatic breast cancer patients. Breast Cancer Res.

[208] Kasimir-Bauer S, Hoffmann O, Wallwiener D, Kimmig R, Fehm T (2012). Expression of stem cell and epithelial-mesenchymal transition markers in primary breast cancer patients with circulating tumor cells. Breast Cancer Res.

[209] Tate JG, Bamford S, Jubb HC, Sondka Z, Beare DM, Bindal N (2019). COSMIC: the Catalogue Of Somatic Mutations In Cancer. Journal.

[210] Dunlap J, Le C, Shukla A, Patterson J, Presnell A, Heinrich MC (2010). Phosphatidylinositol-3-kinase and AKT1 mutations occur early in breast carcinoma. Breast Cancer Res Treat.

[211] Soung YH, Lee JW, Nam SW, Lee JY, Yoo NJ, Lee SH (2006). Mutational analysis of AKT1, AKT2 and AKT3 genes in common human carcinomas. Oncology.

[212] Lopez-Cortes A, Leone PE, Freire-Paspuel B, Arcos-Villacis N, Guevara-Ramirez P, Rosales F (2018). Mutational Analysis of Oncogenic AKT1 Gene Associated with Breast Cancer Risk in the High Altitude Ecuadorian Mestizo Population. Biomed Res Int.

[213] Banerji S, Cibulskis K, Rangel-Escareno C, Brown KK, Carter SL, Frederick AM (2012). Sequence analysis of mutations and translocations across breast cancer subtypes. Nature.

[214] Bleeker FE, Felicioni L, Buttitta F, Lamba S, Cardone L, Rodolfo M (2008). AKT1(E17K) in human solid tumours. Oncogene.

[215] Brugge J, Hung M-C, Mills GB (2007). A new mutational AKTivation in the PI3K pathway. Cancer Cell.

[216] Li G, Guo X, Chen M, Tang L, Jiang H, Day JX (2018). Prevalence and spectrum of AKT1, PIK3CA, PTEN and TP53 somatic mutations in Chinese breast cancer patients. PLoS ONE.

[217] Kadota M, Sato M, Duncan B, Ooshima A, Yang HH, Diaz-Meyer N (2009). Identification of novel gene amplifications in breast cancer and coexistence of gene amplification with an activating mutation of PIK3CA. Cancer Res.

[218] Stephens PJ, Tarpey PS, Davies H, Van Loo P, Greenman C, Wedge DC (2012). The landscape of cancer genes and mutational processes in breast cancer. Nature.

[219] Troxell ML, Levine J, Beadling C, Warrick A, Dunlap J, Presnell A (2010). High prevalence of PIK3CA/AKT pathway mutations in papillary neoplasms of the breast. Mod Pathol.

[220] Yi KH, Axtmayer J, Gustin JP, Rajpurohit A, Lauring J (2013). Functional analysis of non-hotspot AKT1 mutants found in human breast cancers identifies novel driver mutations: implications for personalized medicine. Oncotarget.

[221] Parikh C, Janakiraman V, Wu WI, Foo CK, Kljavin NM, Chaudhuri S (2012). Disruption of PH-kinase domain interactions leads to oncogenic activation of AKT in human cancers. Proc Natl Acad Sci U S A.

[222] Mosquera JM, Varma S, Pauli C, MacDonald TY, Yashinskie JJ, Varga Z (2015). MAGI3-AKT3 fusion in breast cancer amended. Nature.

[223] Meric-Bernstam F, Frampton GM, Ferrer-Lozano J, Yelensky R, Perez-Fidalgo JA, Wang Y (2014). Concordance of genomic alterations between primary and recurrent breast cancer. Mol Cancer Ther.

[224] Liu J, Wei XL, Huang WH, Chen CF, Bai JW, Zhang GJ (2012). Cytoplasmic Skp2 expression is associated with p-Akt1 and predicts poor prognosis in human breast carcinomas. PLoS ONE.

[225] Jordan NJ, Gee JMW, Barrow D, Wakeling AE, Nicholson RI (2004). Increased constitutive activity of PKB/Akt in tamoxifen resistant breast cancer MCF-7 cells. Breast Cancer Res Treat.

[226] Liang K, Lu Y, Li X, Zeng X, Glazer RI, Mills GB (2006). Differential roles of phosphoinositide-dependent protein kinase-1 and akt1 expression and phosphorylation in breast cancer cell resistance to Paclitaxel, Doxorubicin, and gemcitabine. Mol Pharmacol.

[227] Knuefermann C, Lu Y, Liu B, Jin W, Liang K, Wu L (2003). HER2/PI-3K/Akt activation leads to a multidrug resistance in human breast adenocarcinoma cells. Oncogene.

[228] Sokolosky ML, Stadelman KM, Chappell WH, Abrams SL, Martelli AM, Stivala F (2011). Involvement of Akt-1 and mTOR in sensitivity of breast cancer to targeted therapy. Oncotarget.

[229] Taylor JR, Lehmann BD, Chappell WH, Abrams SL, Steelman LS, McCubrey JA (2011). Cooperative effects of Akt-1 and Raf-1 on the induction of cellular senescence in doxorubicin or tamoxifen treated breast cancer cells. Oncotarget.

[230] Fohlin H, Pérez-Tenorio G, Fornander T, Skoog L, Nordenskjöld B, Carstensen J (2013). Akt2 expression is associated with good long-term prognosis in oestrogen receptor positive breast cancer. Eur J Cancer.

[231] Zhou G-L, Tucker DF, Bae SS, Bhatheja K, Birnbaum MJ, Field J (2006). Opposing roles for Akt1 and Akt2 in Rac/Pak signaling and cell migration. J Biol Chem.

[232] Linnerth-Petrik NM, Santry LA, Moorehead R, Jücker M, Wootton SK, Petrik J (2016). Akt isoform specific effects in ovarian cancer progression. Oncotarget.

[233] Pal K, Cao Y, Gaisina IN, Bhattacharya S, Dutta SK, Wang E (2014). Inhibition of GSK-3 induces differentiation and impaired glucose metabolism in renal cancer. Mol Cancer Ther.

[234] Cohen P, Frame S (2001). The renaissance of GSK3. Nat Rev Mol Cell Biol.

[235] Gonzalez E, McGraw TE (2009). The Akt kinases: isoform specificity in metabolism and cancer. Cell Cycle.

[236] Lim C-Y, Bi X, Wu D, Kim JB, Gunning PW, Hong W (2015). Tropomodulin3 is a novel Akt2 effector regulating insulin-stimulated GLUT4 exocytosis through cortical actin remodeling. Nat. Commun.

[237] Cristiano BE, Chan JC, Hannan KM, Lundie NA, Marmy-Conus NJ, Campbell IG (2006). A specific role for AKT3 in the genesis of ovarian cancer through modulation of G(2)-M phase transition. Cancer Res.

[238] Virtakoivu R, Pellinen T, Rantala JK, Perila M, Ivaska J (2012). Distinct Roles of AKT Isoforms in Regulating Beta1-integrin Activity, Migration and Invasion in Prostate Cancer. Eur J Cancer.

[239] Toker A, Yoeli-Lerner M (2006). Akt signaling and cancer: Surviving but not moving on. Cancer Res.

[240] Gao F, Alwhaibi A, Sabbineni H, Verma A, Eldahshan W, Somanath PR (2017). Suppression of Akt1-beta-catenin pathway in advanced prostate cancer promotes TGFbeta1-mediated epithelial to mesenchymal transition and metastasis. Cancer Lett.

[241] Alwhaibi A, Verma A, Artham S, Adil MS, Somanath PR (2019). Nodal pathway activation due to Akt1 suppression is a molecular switch for prostate cancer cell epithelial-to-mesenchymal transition and metastasis. Biochem Pharmacol.

[242] Rao G, Pierobon M, Kim IK, Hsu WH, Deng J, Moon YW (2017). Inhibition of AKT1 signaling promotes invasion and metastasis of non-small cell lung cancer cells with K-RAS or EGFR mutations. Sci Rep.

[243] Malanga D, De Marco C, Guerriero I, Colelli F, Rinaldo N, Scrima M (2015). The Akt1/IL-6/STAT3 pathway regulates growth of lung tumor initiating cells. Oncotarget.

[244] Chen L, Kang QH, Chen Y, Zhang YH, Li Q, Xie SQ (2014). Distinct roles of Akt1 in regulating proliferation, migration and invasion in HepG2 and HCT 116 cells. Oncol Rep.

[245] Cariaga-Martinez AE, Lopez-Ruiz P, Nombela-Blanco MP, Motino O, Gonzalez-Corpas A, Rodriguez-Ubreva J (2013). Distinct and specific roles of AKT1 and AKT2 in androgen-sensitive and androgen-independent prostate cancer cells. Cell Signal.

[246] Lee MW, Kim DS, Lee JH, Lee BS, Lee SH, Jung HL (2011). Roles of AKT1 and AKT2 in non-small cell lung cancer cell survival, growth, and migration. Cancer Sci.

[247] Häggblad Sahlberg S, Mortensen AC, Haglöf J, Engskog MKR, Arvidsson T, Pettersson C (2017). Different functions of AKT1 and AKT2 in molecular pathways, cell migration and metabolism in colon cancer cells. Int J Oncol.

[248] Meng Q, Xia C, Fang J, Rojanasakul Y, Jiang BH (2006). Role of PI3K and AKT specific isoforms in ovarian cancer cell migration, invasion and proliferation through the p70S6K1 pathway. Cell Signal.

[249] Mende I, Malstrom S, Tsichlis PN, Vogt PK, Aoki M (2001). Oncogenic transformation induced by membrane-targeted Akt2 and Akt3. Oncogene.

[250] Dillon RL, Muller WJ (2010). Distinct biological roles for the akt family in mammary tumor progression. Cancer Res.

[251] Yoeli-Lerner M, Toker A (2006). Akt/PKB signaling in cancer: a function in cell motility and invasion. Cell Cycle.

[252] Clark AR, Toker A (2014). Signalling specificity in the Akt pathway in breast cancer. Biochem Soc Trans.

[253] Toker A (2012). Achieving specificity in Akt signaling in cancer. Adv Biol Regul.

[254] Kim EK, Tucker DF, Yun SJ, Do KH, Kim MS, Kim JH (2008). Linker region of Akt1/protein kinase Balpha mediates platelet-derived growth factor-induced translocation and cell migration. Cell Signal.

[255] Laine J, Kunstle G, Obata T, Noguchi M (2002). Differential regulation of Akt kinase isoforms by the members of the TCL1 oncogene family. J Biol Chem.

[256] Walz HA, Shi X, Chouinard M, Bue CA, Navaroli DM, Hayakawa A (2010). Isoform-specific regulation of Akt signaling by the endosomal protein WDFY2. J Biol Chem.

[257] Girardi C, James P, Zanin S, Pinna LA, Ruzzene M (2014). Differential phosphorylation of Akt1 and Akt2 by protein kinase CK2 may account for isoform specific functions. Biochim Biophys Acta.

[258] Wani R, Qian J, Yin L, Bechtold E, King SB, Poole LB (2011). Isoform-specific regulation of Akt by PDGF-induced reactive oxygen species. Proc Natl Acad Sci U S A.

[259] Akhtar N, Jabeen I (2016). A 2D-QSAR and Grid-Independent Molecular Descriptor (GRIND) Analysis of Quinoline-Type Inhibitors of Akt2: Exploration of the Binding Mode in the Pleckstrin Homology (PH) Domain. PLoS ONE.

[260] Hennessy BT, Smith DL, Ram PT, Lu Y, Mills GB (2005). Exploiting the PI3K/AKT pathway for cancer drug discovery. Nat Rev Drug Discov.

